# Nanog mediated by FAO/ACLY signaling induces cellular dormancy in colorectal cancer cells

**DOI:** 10.1038/s41419-022-04606-1

**Published:** 2022-02-17

**Authors:** Meng Zhang, Ruyi Peng, Haizhou Wang, Zhenwei Yang, Hailin Zhang, Yangyang Zhang, Meng Wang, Hongling Wang, Jun Lin, Qiu Zhao, Jing Liu

**Affiliations:** 1 Department of Gastroenterology, Zhongnan Hospital of Wuhan University, Wuhan, 430071 China; 2 Hubei Clinical Center & Key Lab of Intestinal & Colorectal Diseases, Wuhan, 430071 China

**Keywords:** Colon cancer, Cell growth

## Abstract

Dormant cancer cells drive recurrence and drug resistance, which lead to poor prognosis in colorectal cancer (CRC). The mechanisms that regulate the entry of cancer cells into dormancy remain to be extensively studied. Nanog is a master transcription factor to maintain the self-renewal and pluripotency of stem cells. Since dormant cancer cells are similar to quiescent cancer stem cells, the correlation between dormant state and Nanog in CRC is worth to be explored. Serum deprivation is a common method to establish experimental cellular dormancy model. Here, we verified that serum deprivation-induced CRC cells to enter a cellular dormancy state, characterized by no proliferation, no death, no senescence, resistance to chemotherapy, high expression of dormant markers, metabolic suppression, and recovery to active status. Interestingly, we further identified that Nanog was upregulated in dormant CRC cells. Nanog knockdown could destroy the dormant state of serum-deprived CRC cells while Nanog overexpression could induce dormancy in CRC cells. Mechanistically, Nanog was regulated through a fatty acid oxidation (FAO)/ATP citrate lyase (ACLY)-dependent pathway. FAO increased ACLY expression to promote the synthesis of acetyl-CoA, which was transferred by P300 to accelerate H3K27 acetylation of Nanog promoter. Then, Nanog upregulation increased the transcription of P21 and P27, which promoted the dormancy of CRC cells. Our findings revealed that Nanog could induce cellular dormancy in CRC cells and unlocked a specific mechanism to govern the process.

## Introduction

Colorectal cancer (CRC) is one of the most common malignancies worldwide. Insight into colorectal carcinogenesis and development provides systemic and individual treatment options for CRC patients, such as chemo-radiotherapy, targeted therapy and immunotherapy [1]. Although the average 5-year survival rate of CRC patients has ascended to about 64%, the survival rate of advanced patients is lower than 15% [2]. Worse still, CRC has been reported to be the leading causes of cancer-related death in men aged 20–49 from 2012 to 2016 [3, 4]. Therefore, it is urgent to explore the underlying mechanism for metastasis and recurrence of CRC.

To date, increasing evidences have shown that disseminated tumor cells led to metastasis and recurrence of CRC after a period of dormancy [5]. Dormancy is recognized as a critical biological event for cancer cells surviving in an extremely harsh environment. Tumor cells in a dormant state are nonproliferative, quiescent, and they can escape from chemotherapeutics but can recur growth under certain conditions [5, 6]. For example, a study demonstrated that IFN-γ induced tumor-repopulating cells (TRCs) to enter dormancy to evade immunotherapy, which was mediated by an indolamine 2,3-dioxygenase 1 (IDO1)-kynurenine (Kyn)-aryl hydrocarbon receptor (AhR)-p27 dependent pathway [7]. Recently, the orphan nuclear receptor COUP-TF1 and DEC2 [8, 9], as well as the cell cycle regulating proteins P21 and P27 [10] have been identified as tumor cell dormancy markers. In addition to these molecular markers, cellular dormancy can be functionally defined as G0/G1 arrest, being neither dead nor senescent, consuming less glucose, metabolic suppression, decreasing responses to stimulation, and regrowth once the dormancy stimuli are removed [11]. Thus, exploring the detailed mechanisms of dormancy may provide novel therapeutic approaches for CRC treatment.

Nanog is a master transcription factor to maintain the self-renewal and pluripotency of stem cells [12]. Accumulated evidences have demonstrated that Nanog was dysregulated in human cancers, such as colorectal carcinoma, hepatocellular carcinoma, breast and ovarian carcinomas [13]. Functional studies showed that Nanog played a vital role in proliferation, tumorigenicity, invasiveness, stem cell-like property and therapeutic resistance of tumor cells [12]. Our previous study has shown that Nanog promoted the growth and self-renewal of colorectal cancer stem cells [14]. Dormant cancer cells are similar to quiescent cancer stem cells, in terms that they are both cancer cells in G0 phase [15]. Another study has suggested that pluripotency-associated transcription factors SOX2, SOX9, Nanog, and RARB could be transcriptional regulators of dormancy in head and neck, prostate and breast cancers [9, 16, 17]. Therefore, it could be interesting to explore the relationship between cellular dormancy and Nanog in CRC cells.

The experimental cellular dormancy models, such as nutrient deprivation, hypoxia induced were commonly used [18]. Here, we showed that serum deprivation-induced CRC cells to enter a cellular dormancy state. The, Nanog expression were analyzed in dormant CRC cells. The role of Nanog on cellular dormancy as well as its regulating mechanism were further explored. Our findings proposed a novel signaling pathway and potential therapeutic targets to overcome CRC.

## Materials and methods

### Cell lines and cell culture

Human CRC cell line HCT116 and HT29 were bought from China Center for Type Culture Collection (Wuhan, CN). Cell lines were recently authenticated by STR profiling and tested for mycoplasma contamination using TransDetect® PCR Mycoplasma Detection Kit (TransGen Biotech, Peking, CN). Cells were cultured on the rigid dish with McCoy’s 5 A Medium (Hyclone, Utah, USA) containing 10% fetal bovine serum (FBS) (Gibco, Grand Island, USA) and 1% Penicillin-Streptomycin (Hyclone, USA) in 37 °C with 5% CO_2_. Cells were passaged with 0.25% Trypsin (Servicebio, Wuhan, CN) every 3–4 days.

### Reagents

Dimethyl sulfoxide (DMSO), FAO activator bezafibrate (BZF) and inhibitor etomoxir (ETO), glycolysis inhibitor 2-Deoxy-D-glucose (2-DG), glutaminase inhibitor BPTES, P300 inhibitor C646 and 5-fluorouracil (5-FU) were all purchased from MedChemExpress, New Jersey, USA.

### Semi-quantitative PCR

Total RNA of cells was extracted with Trizol reagent (Invitrogen, California, USA) according to the instruction manual. Reverse transcription (RT) was conducted with RevertAid First Strand cDNA Synthesis Kit (Thermo, Massachusetts, USA). PCR was performed using 2×Taq PCR StarMix with Loading Dye (GenStar, Peking, CN). The grey value was analyzed by Image J. Data were analyzed with the comparative grey value method for relative gene-expression quantification against GAPDH. Primers were designed and synthesized by the Tsingke Company (Peking, CN) and shown in Table S1.

### Western blotting

Western blotting was performed as previously described [14]. The primary antibodies used in this study included: GAPDH (Proteintech #10494-1-AP, Wuhan, CN), Nanog (Proteintech #14295-1-AP, CN), ACLY (Proteintech #15421-1-AP, CN), P300 (Cell Signaling Technology #86377, Boston, USA), COUP-TF1 (Proteintech #24573-1-AP, CN), hDEC (Proteintech #212688-1-AP, CN), P21 (Proteintech #10355-1-AP, CN), P27 (Proteintech #25614-1-AP, CN) and H3K27Ac (Cell Signaling Technology #8173, USA). The secondary antibody Anti-rabbit IgG-HRP (Servicebio #GB23303, CN) was purchased from Servicebio, CN. All grey values including the GAPDH were quantified by Image J, and the relative expression was calculated by comparing to the control groups and GAPDH. Results were confirmed by at least three independent experiments.

### Calcein-AM/PI double staining

CRC cells were cultured in normal condition or in serum-free condition for 12 h or 24 h. Calcein-AM/propidium iodide (PI) double stain kit (KeyGEN BioTECH, Nanjing, CN) was then used to detect cell death following the manufacturer’s manual. In brief, the working solution containing Calcein-AM and PI (1:4) was prepared. Cells were washed 3 times with PBS and incubated with the working solution in 37 °C for 45 min. Images were acquired from fluorescence microscope (Olympus, Tokyo, Japan).

### Cell cycle analysis

Cells were incubated with 10 mM EdU (RIBOBIO, Guangzhou, CN) for 2 h, then stained with anti-EdU antibody (RIBOBIO, CN) and 5 mg/ml of PI (KeyGen Biotech, CN). The samples were analyzed by flow cytometry (BD Biosciences, New Jersey, USA). The cell cycle distribution was determined as the percentage of the total population: G0-G1 (2n, EdU negative), S (2n- 4n, EdU positive) and G2 phase (4n, EdU negative).

### Apoptosis analysis

CRC cells were cultured in normal condition or in serum-free condition for 12 h. In some experiments, cells were then treated with 5-FU in 150 μg/ml for 18 h. After that, cells were collected for apoptosis analysis with the process described before [14].

### Measurement of glucose concentration

CRC cells were cultured in normal condition or serum-free condition, the culture medium was collected after 24 h. Glucose concentration in the culture medium was measured using Glucose detecting kit (RSBio, Shanghai, CN). Glucose consumption = the primary glucose concentration- the terminal glucose concentration.

### Cell Viability Assay

CRC cells were seeded in 96-well plates with a density of 2000 cells/well in 100 μL culture medium, incubated overnight and treated as indicated. The cell viability was detected with cck8 kit (Biosharp life sciences, Hefei, CN).

### SA-β-gal activity assay

CRC cells were fixed at room temperature for 15 min, then stained overnight with the β-Galactosidase Staining Kit (Cell Signaling Technology, MA, USA) at 37°C without CO_2_. Images were taken under white light using a microscope (Olympus, Japan).

### Chromatin immunoprecipitation (ChIP)

Specific anti-human Nanog antibody (Cell Signaling Technology #3580, USA) and anti-human H3K27Ac antibody (Cell Signaling Technology #8173, USA) were used for immunoprecipitation studies. ChIP was performed with the SimpleChIP® Enzymatic Chromatin IP Kit (Cell Signaling Technology, USA). Cell sonication was performed with the AVCX130 system (Sonics & Materials, Newtown, USA). Anti-Nanog, anti-H3K27Ac and normal anti-human-IgG (Cell Signaling Technology #3900, USA) antibodies were applied for immunoprecipitation reactions. Semi-quantitative PCR was used to amplify the DNA fragments recovered from immunoprecipitated complexes. The primer sequences were shown in Table S2.

### Oil red O staining

Cells were washed with PBS three times and fixed for 30 min with 4% paraformaldehyde, then washed with PBS two times and dyed with oil red O solution (Biovision, California, USA) for 30 min at room temperature. After that, cells were immersed in 60% isopropanol for 5 sec and immediately rinsed with PBS 3 times. Cells were observed and photographed with the microscope.

### Plasmids and siRNA transfection

Empty vector pCMV3 and pCMV3-Nanog plasmids were purchased from Sino Biological Inc., Peking, CN. Using Lipofectamine 2000 (Invitrogen, USA), HT29 and HCT116 cells were transfected with pCMV3-Nanog or negative control vector (2.5 μg per well) following the manufacturer’s instruction. After 48 h, cells were collected or in some experiments, continuously cultured in serum deprivation condition for the indicated time.

Nanog siRNAs, ACLY siRNAs and negative control siRNAs were purchased from Ribobio (Guangzhou, CN). Corresponding siRNAs (50 nM) were delivered to HT29 and HCT116 cells. After 48 h, CRC cells were cultured in serum deprivation condition for the indicated time. The siRNA sequences are provided in Table S3.

### Immunohistochemistry (IHC) and immunofluorescence (IF) staining

Paraffin-cut sections of tumors were prepared. The sections were dewaxed, rehydrated, and antigen retrieved in turn, followed by a blocking step in 5% BSA for 40 min. Then incubated with primary antibodies Nanog (Servicebio #GB11331, CN), CD31 (Servicebio #GB11063, CN) at 4 °C overnight, with anti-rabbit secondary antibody labeled with HRP (Servicebio #GB23303, CN) for 2 h. Images were taken by fluorescence microscope (Olympus, Japan).

Cells grew on the rigid dish were washed 3 times with PBS and fixed for 10 min with 4% paraformaldehyde. After permeabilizing with 0.1% Triton-X-PBS and blocking in 1% BSA-PBS, the cells were incubated for 1 h with primary antibodies: COUP-TF1 (Proteintech #24573-1-AP, CN), hDEC (Proteintech #212688-1-AP, CN), Nanog (Proteintech #14295-1-AP, CN), or Ki67 (Proteintech #27309-1-AP, CN), washed with PBS for 3 times, incubated with CY3-conjugated anti-rabbit secondary antibody (Servicebio #G1223, CN) for 45 min, and washed with PBS for 3 times. nuclei were marked by DAPI (Servicebio #G1012, CN). IF images were taken with fluorescence microscope (Olympus, Japan).

### Colon cancer samples

6 colon cancer samples were obtained from the Zhongnan Hospital of Wuhan University. Informed consent was gotten from the patients. Approval was acquired for procedures involved in human subjects from the Ethics Committee of the Zhongnan Hospital of Wuhan University. The privacy rights of human subjects were observed. IF was performed as above.

### Animal experiments

Animal experiments were approved by the Animal Ethics Committee of Wuhan University and conducted under standard procedures. BALB/c nude mice (6–8 weeks) were purchased from Beijing Vital River Laboratory Animal Technology Company (CN). 6 female mice were strictly and equally randomized to two groups, experimenters were blinded from the treatment paradigm and where not involved in data analysis. 1 × 10^5^ HT29 or HCT116 cells were subcutaneously injected into the right flank. Mice were sacrificed through cervical vertebra dislocation after 4 weeks. Tumors were dissected fixed in 37% formalin. IHC was performed as above.

### Statistical analysis

Data were analyzed by GraphPad 8.0 software (GraphPad Software Inc., California, USA). Before statistical analysis, the homogeneity of variance between groups was tested. Statistical significance between groups was analyzed using Two tailed Student’s *t*-test, all data followed a normal distribution. *P < 0.05, **P < 0.01, ***P < 0.001. *P* < 0.05 was considered as significant difference. All data represented multiple independent experiments unless otherwise indicated.

## Results

### Serum deprivation induces cellular dormancy of CRC cells in vitro

**Fig. 1:**
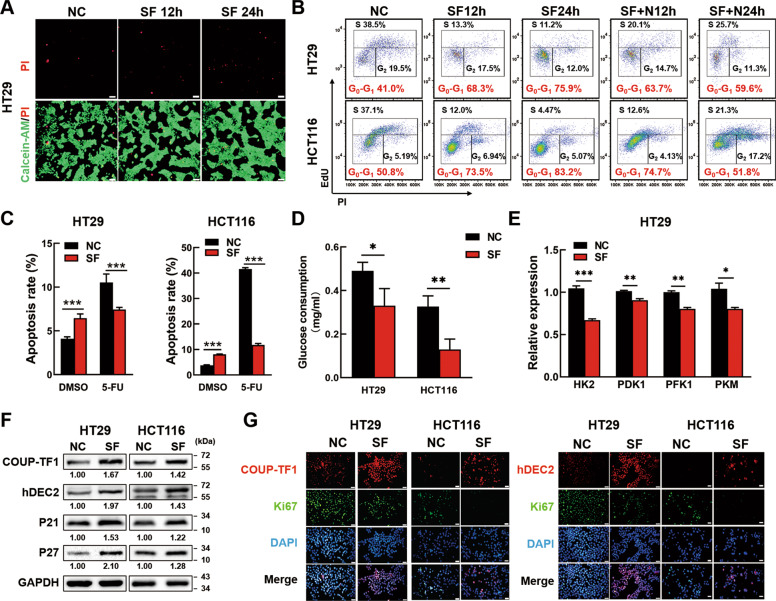
Serum deprivation induces CRC cell dormancy in vitro. **A** Calcein-AM/PI staining of serum-deprived CRC cells. HT29 cells were cultured in serum-free conditions for 12 h (SF12h) or 24 h (SF12h). Cells in normal conditions (NC) were used as controls. Living cells were stained by Calcein-AM (green) while dead cells were visualized by PI (red). The stained cells were observed under a fluorescence microscope and photographed. Scale bar = 50 μm. **B** Reversible G0-G1 arrest in CRC cells. CRC cells were cultured in normal condition (NC), serum withdrawal for 12h (SF12h) or 24 h (SF24h), Serum replenishment for 12 h (SF + N12h) or 24 h (SF + N24h) after serum withdrawal 24 h, respectively. The cell cycle was analyzed by FCM. **C** Serum deprivation protected colon cells from chemotherapeutic apoptosis. CRC cells were serum deprived for 24h before treating with 150 μg/ml 5-FU for 18 h. Then, the cells were tested for apoptosis by FCM. **D** SF stimulation reduced the glucose consumption of CRC cells. HT29 and HCT116 CRC cells were cultured in normal condition or serum-free condition, the culture medium was collected after 24 h. Glucose concentration in the culture medium was measured using Glucose detecting kit and glucose consumption was calculated. **E** The mRNA expression of genes (*HK2*, *PDK1*, *PFK1* and *PFM*) involved in glucose catabolism in NC and SF HT29 cells was analyzed by PCR. **F** The protein expression of COUP-TF1, hDEC2, P21 and P27 in NC and SF CRC cells was analyzed by Western blotting. The relative expression was quantified by Image J. **G** Immunofluorescence staining of COUP-TF1 or hDEC2 and Ki67 from serum-deprived CRC cells or normal cells. Scale bar = 20 μm. Data are shown as the means ± s.e.m., *n* = 3; *P < 0.05, **P < 0.01, ***P < 0.001. NC normal condition, SF: serum deprivation for 24 h; FCM Flow cytometry. Data represented at least 3 independent experiments.

To establish the cellular dormancy model in CRC cells, human CRC cell line HT29 and HCT116 were cultured in serum-free conditions (SF) or normal conditions (NC) with 10% fetal bovine serum for the indicated time. Firstly, cell cycle was analyzed in two different culture conditions by Flow cytometry. Data showed that serum deprivation could induce G0-G1 cell cycle arrest from day 1 to day 5, while the cell number decreased obviously at day 7 (Fig. S1). Then, cell viability was analyzed by CCK8. Data showed that cell viability of normal cultured CRC cells kept raising within 96 h, but for SF CRC cells, the cell viability showed no significant change within 72 h and dropped significantly from 72 h to 96 h (Fig. S2A). It meant that SF could induce CRC cells to a nonproliferative state as short as 24 h. Therefore, we decided to further study the dormant features of serum-deprived CRC cells within 24 h. Data showed that few CRC cells were dead under serum deprivation for 12 h and 24 h by Calcein-AM/PI staining (Fig. 1A, Fig. S2B). Cell cycle experiments further showed CRC cells underwent G0-G1 cell cycle arrest since serum deprived for 12 h and aggravated at 24 h (Fig. 1B). Even more to the point, the percentage of SF CRC cells in G0-G1 phase remarkably decreased when recovered serum supply for 12 or 24 h (Fig. 1B), which indicated that the cell cycle arrest in SF CRC cells could be rescued. In addition, SF CRC cells did not undergo senescence as evaluated by SA-β-gal activity assay (Fig. S2C). Meanwhile, in the group SF CRC cells treated with the chemotherapeutics 5-fluorouracil (5-FU), the apoptosis rate of which was significantly lower than that of NC cells treated with 5-FU, while the tendency was contrary to that in DMSO group (Fig. 1C). It suggested that although serum deprivation could induce slightly apoptosis of CRC cells, SF CRC cells were resistant to 5-FU induced apoptosis. We also found SF CRC cells consumed less glucose (Fig. 1D) and exhibited lower mRNA expression of genes involved in glucose catabolism than NC CRC cells (Fig. 1E, Fig. S2D). Taken together, SF CRC cells showed a state of revisable cell cycle arrest, neither death nor senescence, consuming less glucose and resistance to chemotherapy, which indicated that SF CRC cells entered a cellular dormancy state.

To further validate, we examined the dormancy biomarkers (e.g. orphan nuclear receptor COUP-TF1 and hDEC2) and cell cycle-regulatory molecules (e.g. P21 and P27). The PCR and Western blotting results showed that serum deprivation upregulated the expression of COUP-TF1, hDEC2, P21 and P27 significantly in CRC cells at both mRNA and protein levels (Fig. S3A, Fig. 1F). In line with the cell cycle arrest rescue, recovering serum supply could also reduce the protein expression of COUP-TF1, hDEC2, P21 and P27 in SF CRC cells (Fig. S3B). In addition, we stained CRC cells with the proliferation marker Ki67 and COUP-TF1 or hDEC2 by IF. Data showed that SF treatment remarkably increased the percentage of COUP-TF1^+^Ki67^−^ cells or hDEC2^+^Ki67^−^ cells (Fig. 1G). Collectively, these data suggested that serum deprivation for 24 h was capable of inducing CRC cells into cellular dormancy in vitro.

### Nanog regulates cellular dormancy of CRC cells

**Fig. 2:**
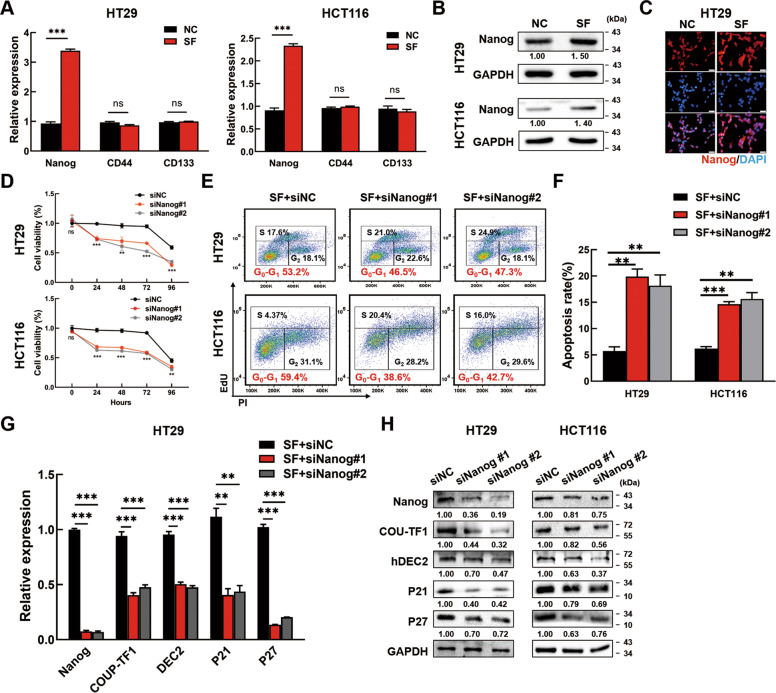
Nanog regulates CRC cell dormancy. **A** The mRNA expression of *Nanog*, *CD44* and *CD133* in NC and SF CRC cells by PCR. **B**, **C** Nanog expression in NC and SF CRC cells was tested by Western blotting (**B**) and Immunofluorescence staining (**C**). Scale bar = 20 μm. **D** Silencing Nanog decreased the cell viability of SF CRC cells. CRC cells were transfected with Nanog siRNAs or negative control siRNAs for 48 h before serum deprivation. Cell viability was detected by CCK8 and marked as SF 0–96 h. **E**, **F** Silencing Nanog relieved the G0-G1 arrest **(E)** and increased the apoptosis rate **(F)** in SF CRC cells. CRC cells were transfected with Nanog siRNAs or negative control siRNAs for 48 h, and serum deprived for 12 h successively, then collected to analyze in the FCM. **G**, **H** Nanog knockdown decreased the expression of COUP-TF1, hDEC2, P21 and P27. Data are shown as the means ± s.e.m., *n* = 3; ns: no statistical significance, **P < 0.01, ***P < 0.001. Data represented at least 3 independent experiments.

It was demonstrated that pluripotency transcription factors could induce dormancy [19]. Therefore, we analyzed the expression of pluripotency transcription factor Nanog, SOX2, OCT4, and CRC stem cell marker CD133, CD44 in SF and NC conditions. PCR showed that Nanog was upregulated significantly in both HT29 and HCT116 SF cells, while the differences of CD133 and CD44 showed no significance (Fig. 2A). Meanwhile, OCT4 and SOX2 expression were almost undetectable in SF CRC cells (Data not shown). Western blotting and IF showed that Nanog protein expression was also enhanced in SF condition and then reduced when serum came back (Fig. 2B & C, Fig. S4A & B). To explore the effects of Nanog on dormancy, Nanog expression was silenced by Nanog siRNAs in SF CRC cells. Firstly, we transfected SF CRC cells with Nanog siRNAs or NC siRNAs, which were then cultured in SF conditions for another 96 h. CCK8 assays were performed to analyze the cell viability. The results showed that silencing Nanog reduced the cell viability (Fig. 2D). In addition, Nanog knockdown decreased the percentage of SF CRC cells in G0-G1 phase (Fig. 2E) and increased the apoptosis rate of SF CRC cells (Fig. 2F). PCR showed that silencing Nanog inhibited the mRNA expression of COUP-TF1, hDEC2, P21 and P27 (Fig. 2G, Fig. S4C). Accordantly, Western blotting and IF showed that silencing Nanog inhibited the protein levels of COUP-TF1, hDEC2, P21 and P27 (Fig. 2H, Fig. S4D). The above results manifested that knockdown of Nanog could destroy the dormant state of SF CRC cells.

**Fig. 3:**
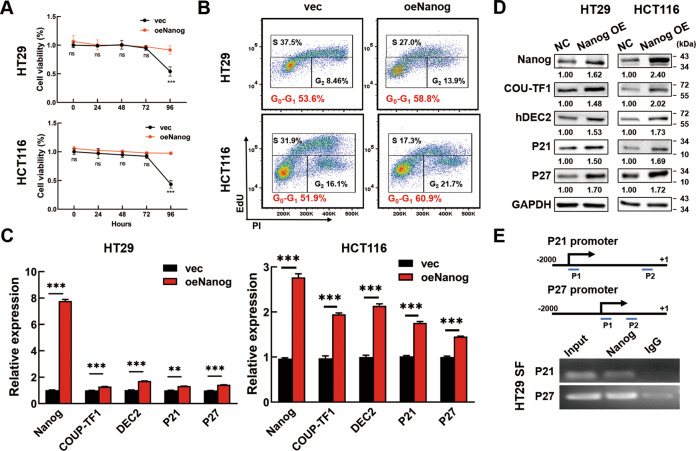
Nanog overexpression induces CRC cell dormancy. **A** Nanog overexpression increased the cell viability of SF CRC cells. CRC cells were transfected with pCMV3-Nanog plasmids or empty vector for 48 h before serum deprivation. Cell viability was detected by CCK8 and marked as SF 0–96 h. **B** Nanog overexpression increased the percentage of CRC cells in G0-G1 phase. CRC cells were transfected with pCMV3-Nanog plasmids or empty vector for 48 h before analyzing in FCM. **C**, **D** Nanog overexpression increased the expression of COUP-TF1, hDEC2, P21 and P27. CRC cells were transfected with pCMV3-Nanog plasmids for 48 h. The empty vector pCMV3 plasmids were used as controls. The expression of COUP-TF1, hDEC2, P21, and P27 was tested by PCR and Western blotting. The relative expression was quantified by Image J. **E** SF HT29 cells were collected for ChIP-PCR with anti-Nanog antibody and specific primers for P21 and P27. IgG was used as negative control. Scheme of the different positions of ChIP-PCR primers was also shown (P21-p1: −1366/−1347 bp, P21-p2: −420/−399 bp, P27-p1: −977/ −958 bp, P27-p2: −698/−679 bp). Data are shown as the means ± s.e.m., *n* = 3; **P < 0.01, ***P < 0.001. Data represented at least 3 independent experiments.

On the other hand, Nanog was overexpressed in CRC cells by Nanog plasmids to verify the effects of Nanog on dormancy. CCK8 assays showed that overexpression of Nanog could extend the nonproliferative state to 96 h for SF CRC cells (Fig. 3A). At the same time, overexpressing Nanog increased the percentage of CRC cells in G0-G1 phase (Fig. 3B). Moreover, Nanog overexpression induced the expression of COUP-TF1, hDEC2, P21, and P27 at both mRNA and protein levels (Fig. 3C, D). These results suggested that Nanog mediated cellular dormancy of SF CRC cells.

To study how Nanog induced dormancy, CHIP-PCR assay was conducted on serum-deprived HT29 cells. Scheme of P21 and P27 promotor were used to show the enrichment of Nanog on different loci. Data showed Nanog could bind to the promotor of both P21 and P27 in the P21-p1 and P27-p1 region (Fig. 3E). The results indicated that Nanog performed as a transcription factor to enhance the expression of P21 and P27, and thus induced the cellular dormancy of CRC cells.

### Nanog expression is mediated by fatty acid oxidation (FAO) to induce cellular dormancy of SF CRC cells

**Fig. 4:**
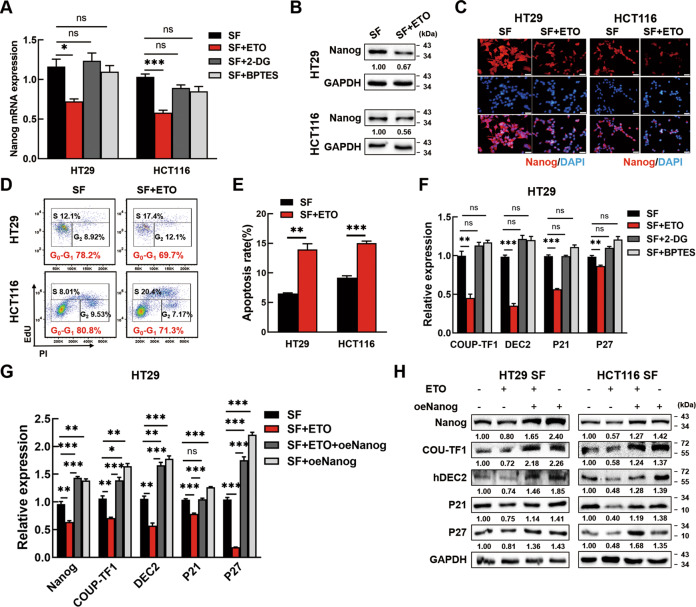
Nanog expression is mediated by fatty acid oxidation (FAO) to induce CRC cell dormancy. **A** ETO could block Nanog expression in serum-deprived CRC cells while 2-DG and BPTES could not. The CRC cells were serum-deprived and treated with 100 μM ETO, 10 μM 2-DG or 10 μM BPTES for 24 h. Then, the cells were tested for *Nanog* expression by PCR. **B**, **C** Nanog expression in serum-deprived CRC cells treated with 100 μM ETO was tested by Western blotting (**B**) and Immunofluorescence staining (**C**). The stained cells were observed with a fluorescent microscope and photographed. Scale bar = 20 μm. **D** ETO treatment decreased the percentage of SF CRC cells in G0-G1 phase. CRC cells were serum deprived and treated with ETO for 24 h before collected to cell cycle analysis. **(E)** ETO treatment increased the apoptosis rate in SF CRC cells. CRC cells were serum deprived and treated with 100 μM ETO for 24 h, concurrently, and then collected to analyze in the FCM. **F** ETO could block the expression of COUP-TF1, hDEC2, P21 and P27 in SF CRC cells while 2-DG and BPTES could not. **G**, **H** The blockage of dormancy markers by ETO could be reversed by Nanog overexpression. ETO: etomoxir, FAO inhibitor; 2-DG: 2-Deoxy-D-glucose, glycolysis inhibitor; BPTES: glutaminase inhibitor. Data are shown as the means ± s.e.m., *n* = 3; ns: no statistical significance, *P < 0.05, **P < 0.01, ***P < 0.001. Data shown represented three independent experiments.

Next, we sought to uncover the mechanism underlying Nanog regulation in serum-deprived CRC cells. Aerobic glycolysis, FAO and glutaminolysis are the main metabolic rewiring features by which cancer cells adapted to nutrition limitation. To identify the potential pathway regulated Nanog expression, FAO inhibitor etomoxir (ETO), glycolysis inhibitor 2-Deoxy-D-glucose (2-DG) or glutaminase inhibitor BPTES was applied in SF CRC cells. PCR and Western blotting showed that only ETO inhibited Nanog expression in SF CRC cells (Fig. 4A, Fig. S5). Western blotting and IF confirmed that the protein levels of Nanog were also decreased after ETO treatment in SF CRC cells (Fig. 4B, C). We also treated normal CRC cells with bezafibrate (BZF), a common FAO agonist, and found that Nanog protein expression was apparently enhanced (Fig. S6A). So far, our data indicated that Nanog upregulation was FAO dependent in dormant CRC cells.

After that, we investigated whether FAO was critical for CRC cell dormancy. Oil red O staining showed significant consumption of lipid droplets in HT29 and HCT116 cells after SF treatment (Fig. S6B). When blocking FAO with ETO, G0-G1 arrest of dormant CRC cells was alleviate (Fig. 4D). When facilitating FAO with BZF, G0-G1 arrest and lessened S phase percentage were observed in normal CRC cells (Fig. S6C). We also found a distinct increase of apoptosis rate in ETO treated SF CRC cells (Fig. 4E). These results indicated FAO was necessary to sustain cellular dormancy of serum-deprived CRC cells.

We further explored the expression of COUP-TF1, DEC2, P21, and P27 in SF CRC cells treated with ETO, 2DG or BPTES respectively. Consistent with the tendency of Nanog, they were all inhibited by ETO but not by 2DG and BPTES (Fig. 4F, Fig. S6D). Meanwhile, the expression of COUP-TF1, DEC2, P21, and P27 were all enhanced in normal CRC cells treated with BZF (Fig. S6E). Next, SF CRC cells were treated with ETO and further transfected Nanog overexpression plasmids. PCR and Western blotting showed that the decreased expression of COUP-TF1, DEC2, P21, and P27 resulted from ETO treatment could be reversed by Nanog overexpression under serum-deprived conditions (Fig. 4G & H, Fig. S6F). Taken together, these above data showed that the dormancy of serum-deprived CRC cells was mediated by FAO dependent Nanog expression.

### ACLY promotes P300/H3K27Ac expression to accelerate acetylation of Nanog promoter in SF CRC cells

**Fig. 5:**
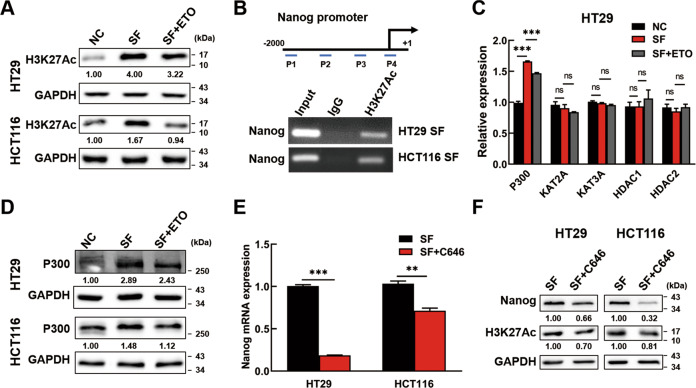
ACLY promotes P300 expression to accelerate H3K27 acetylation of Nanog promoter in SF CRC cells. **A** The expression levels of H3K27Ac were analyzed by Western blotting in normal cells, in SF CRC cells and in SF CRC cells treated with ETO. **B** The interaction of H3K27Ac and Nanog promoter in SF CRC cells was assessed by CHIP-PCR. IgG was used as negative control. Scheme of the different positions of ChIP-PCR primers was also shown (P1: −1844/−823 bp, P2: −1043/−1024 bp, P3: −791/−772 bp, P4: −259/−236 bp). **C** The expression levels of histone acetyltransferases (*P300, KAT2A, KAT3A*) and histone deacetylases (*HDAC1, HDAC2*) were examined by PCR in normal HT29, in SF HT29 cells and in SF HT29 cells treated with ETO. **D** The expression level of P300 was analyzed by Western blotting in normal CRC cells, in serum-deprived CRC cells and in serum-deprived CRC cells treated with ETO. **E**, **F** P300 inhibition downregulated the expression of Nanog and H3K27Ac. The serum-deprived HT29 and HCT116 cells were treated with C646 for 24 h. Then, the cells were tested for Nanog expression by PCR **(E)** and Nanog and H3K27Ac expression by Western blotting **(F)**. C646: P300 inhibitor. Data are shown as the means ± s.e.m., *n* = 3; ns: no statistical significance, **P < 0.01, ***P < 0.001. Data shown represented three independent experiments.

Subsequently, we gained insight into the molecular mechanism of how FAO increases Nanog expression. As the main products of FAO, the effects of acetyl-CoA were taken into account. It was reported that histone acetylation levels were correlate with acetyl-CoA abundance [20]. Thus, we performed western blotting to analyze the histone acetylation levels. In consistent with Nanog expression, H3K27 acetylation (H3K27Ac) was enriched in SF CRC cells and was further inhibited by ETO treatment (Fig. 5A). To further clarify the correlation between Nanog expression and H3K27Ac levels, H3K27Ac was immunoprecipitated by ChIP and its enrichment relative to input chromatin was assessed by PCR with four pairs of primers focusing on the promoter region of Nanog. In SF CRC cells, we found that H3K27Ac accumulated in the promoter of Nanog, especially in region near the transcription starting site (region P4 in the scheme) (Fig. 5B). These data suggested that FAO promoted Nanog expression by amplifying H3K27 acetylation of Nanog promoter.

Histone acetylation is regulated by histone acetyltransferases (HATs) and histone deacetylases (HDACs). The expression of several HATs (P300, KAT2A, KAT3A) and HDACs (HDAC1, HDAC2) was examined. We found that P300 showed the same tendency with Nanog and H3K27Ac (Fig. 5C, Fig. S7). Western blotting further confirmed these results in CRC cells (Fig. 5D). To determine whether P300 regulated H3K27Ac of Nanog promoter in SF CRC cells, a specific inhibitor of P300, C646 was used. Data showed that P300 inhibition could suppress Nanog and H3K27Ac expression (Fig. 5E, F). Therefore, the above data clearly supported that the H3K27 acetylation of Nanog promoter in dormant CRC cells was induced by FAO mediated P300 expression.

**Fig. 6:**
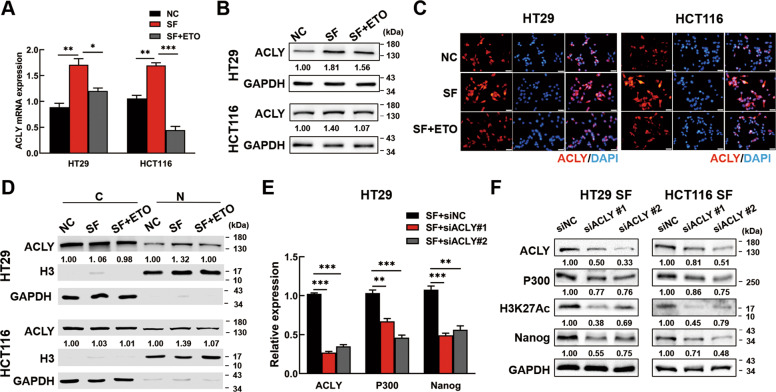
FAO increases nuclear ACLY expression to activate Nanog via P300/H3K27Ac. **A**–**C** The expression of ACLY was analyzed by PCR **(A)**, Western blotting **(B)** and Immunofluorescence staining **(C)** in normal CRC cells, in SF CRC cells and in SF CRC cells treated with ETO. Scale bar = 20 μm. **D** The expression of ACLY in cytoplasm and nucleus was analyzed by Western blotting. **E**, **F** ACLY knockdown decreased the expression of P300 and Nanog. Serum deprived HT29 cells were transfected with ACLY siRNAs or negative control siRNAs. The expression of *ACLY*, *P300* and *Nanog* was analyzed by PCR. **F** Western blotting analysis of P300, H3K27Ac and Nanog expression by ACLY inhibition in serum-deprived CRC cells. Data are shown as the means ± s.e.m., *n* = 3; *P < 0.05, **P < 0.01, ***P < 0.001. Data shown represented three independent experiments.

ATP-citrate lyase (ACLY) is critical for maintaining the pool of acetyl-CoA in the cytoplasm and nucleus, which links cellular metabolism to histone acetylation [20]. To determine if ACLY was the upstream regulator of P300 mediated H3K27Ac in Nanog promoter, PCR, western blotting and IF assays were performed. Data showed that SF condition increased ACLY expression, while ETO treatment almost abolished the effects in CRC cells (Fig. 6A-C). Furthermore, nuclear and cytosolic fractions of the above cells were extracted and analyzed by western blotting. As exhibited, the nuclear ACLY expression were majorly influenced under SF treatment (Fig. 6D). Next, we wonder the effects of ACLY on Nanog regulation. The results showed that P300 and Nanog expression, as well as H3K27 acetylation were downregulated after silencing of ACLY expression (Fig. 6E & F, Fig. S8A). IF further confirmed that Nanog expression and H3K27 Ac were restrained by ACLY knockdown (Fig. S8B). These findings revealed that FAO promoted nuclear ACLY expression to induce acetyl-CoA generation and subsequently transcriptional activated Nanog via P300/H3K27Ac regulation.

### ACLY/P300 mediates Nanog expression to drive cellular dormancy of CRC cells

**Fig. 7:**
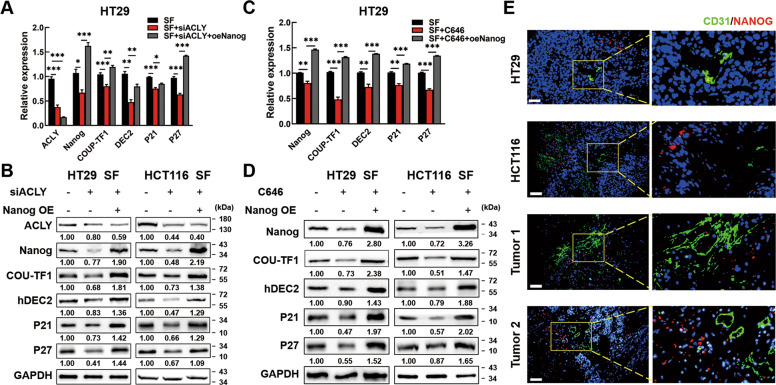
ACLY/P300 mediates Nanog expression to drive CRC cell dormancy. **A**, **B** Dormancy markers could be inhibited by ACLY inhibition and reversed by Nanog overexpression in SF HT29 cells. **C**, **D** Dormancy markers could be inhibited by P300 inhibition and reversed by Nanog overexpression in SF HT29 cells. **E** CD31 (green) and Nanog (red) expression in subcutaneous tumors of mouse and CRC tissues by Immunofluorescence. The stained sections were observed under a fluorescent microscope and photographed. Scale bar = 50 μm. Data are shown as the means ± s.e.m., *n* = 3; *P < 0.05, **P < 0.01, ***P < 0.001. Data shown represented three independent experiments.

**Fig. 8:**
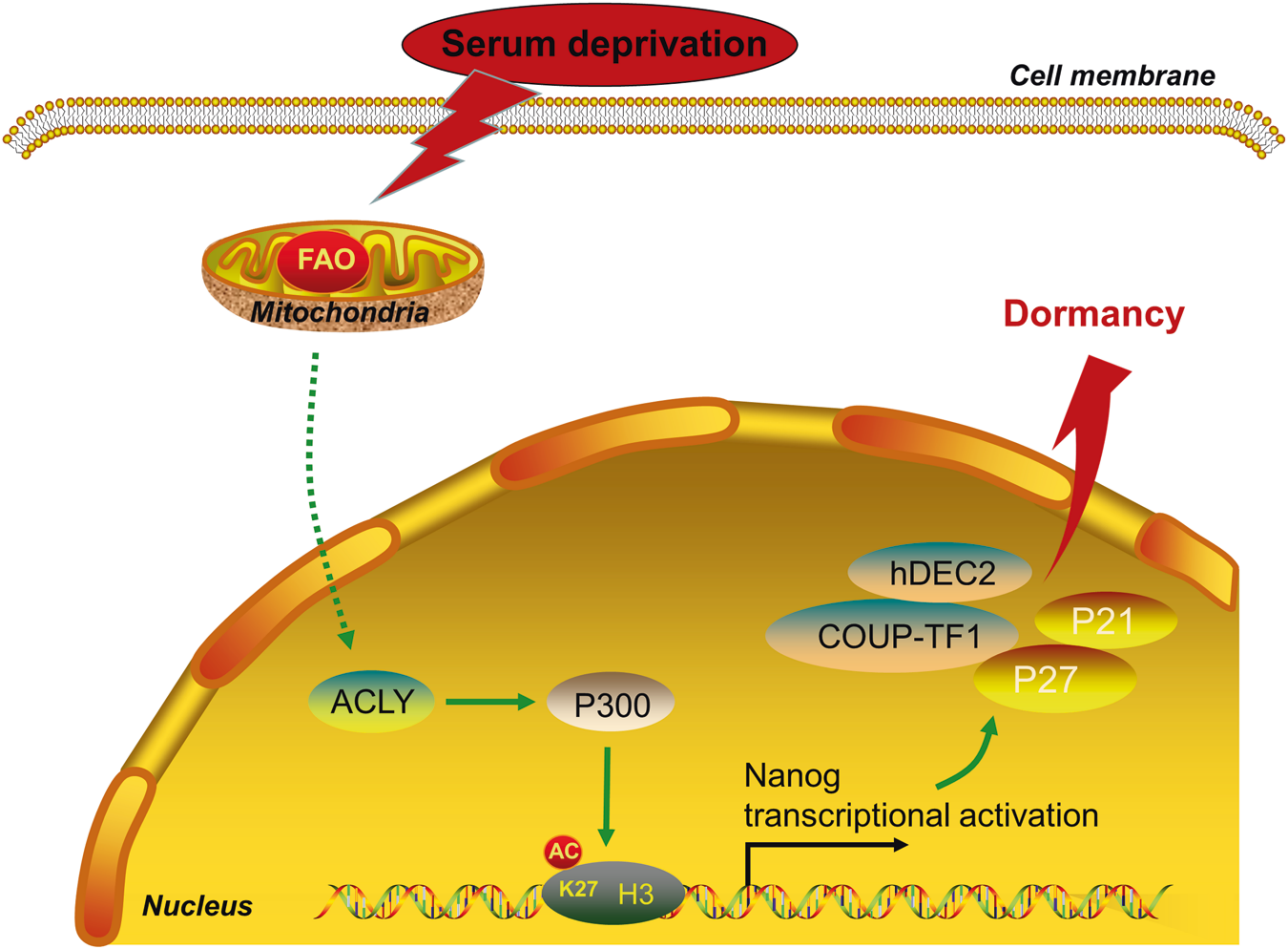
A schematic chart of Nanog-induced dormancy regulatory mechanisms in CRC cells. FAO/ACLY signaling promoted Nanog expression via P300/H3K27Ac regulation. Then, Nanog upregulation increased the transcription of P21, P27,COUP-TF1, and hDEC2, and then induced cellular dormancy of serum deprived CRC cells.

To validate that ACLY regulated Nanog to induce dormancy of SF CRC cells, we inhibited ACLY expression and then transfected with Nanog overexpression plasmids. Data showed that the expression of COUP-TF1, DEC2, P21, and P27 were repressed by silencing ACLY while reactivated by Nanog overexpression under serum-deprived conditions (Fig. 7A & B, Fig. S9A). Similarly, to corroborate that P300 regulated Nanog to induce dormancy of serum-deprived CRC cells, SF CRC cells were treated with C646 and further transfected Nanog overexpression plasmids. Data showed that under serum-deprived conditions, the expression of COUP-TF1 and DEC2 could be down-regulated by P300 inhibition, which were also reversed by Nanog overexpression (Fig. 7C & D, Fig. S9B). To further validate the connection between Nanog expression and dormancy in vivo, we evaluated the CD31 (also called PECAM1), an endothelial marker of blood vessel, and Nanog expression in tumor-bearing mouse and human CRC tissues by IF. We found that Nanog was rarely expressed near blood vessels but expressed more in the poorer nutrition district where far away from blood vessels (Fig. 7E, Fig. S10). Moreover, online datasets showed that Nanog was negatively correlated with CD31 expression in colon cancer tissues by Pearson correlation analysis (Fig. S11). These results partly demonstrated that nutrient deficiency-induced dormancy was positively associated with Nanog expression. The above data showed that ACLY/P300 mediated Nanog expression to drive the dormancy of CRC cells (Fig. 8).

## Discussion

Elucidating the mechanisms of dormancy is of paramount significance. It has been reported that in vitro models mimicking dormant cancer cells can be established through depriving cell cultures of serum, nutrients or oxygen. In the present study, we used serum deprivation to induce dormant CRC cells, characterized by no proliferation, no death, no senescence, resistance to chemotherapy, high expression of dormant markers, metabolic suppression, and recovery to active status. One thing has to be mentioned is that the details of the experimental protocol of serum deprivation to induce dormancy varies in different cell lines. Cellular dormancy could be induced by serum-deprived for 42 h in human multiple myeloma cells or as long as 7 days in prostate cancer cells [21]. Moreover, although most studies involving serum deprivation use the serum-free medium method, low serum media containing 0.05%–0.2% is also employed. Here, we provided an easy model in which serum deprivation for only 24 h could induce cellular dormancy in CRC cells.

Next, we identified that Nanog was upregulated in dormant CRC cells. Nanog knockdown could destroy the dormant state of serum-deprived CRC cells while Nanog overexpression could induce dormancy in CRC cells. Results further showed that Nanog overexpression promoted the expression of P21, P27, COUP-TF1 and hDEC2 to regulate dormancy in CRC cells. Thus, our study complements and extends prior knowledge on Nanog biology in CRC.

The upstream regulatory pathway which mediated Nanog to induce dormancy was then explored. It’s known that nutrient limitation frequently happens during tumor development, and cellular dormancy is one of the important ways to adapt to the harsh conditions for cancer cells [22]. At the same time, numerous studies declared cancer cells could rewire metabolic ways or utilize the additional nutrient sources to conquer kinds of nutrient scarcity, such as Aerobic glycolysis, fatty acid β-oxidation (FAO), and glutaminolysis [23–25]. Recent studies demonstrated that FAO was upregulated in dormant human melanoma tumor repopulating cells and dormant ER + breast cancer cells [10, 26], inhibiting of FAO promoted to clear dormant residual disease, while dietary fat benefited cancer cells survival [26]. Likewise, our data showed that Nanog upregulation was FAO dependent in dormant CRC cells. Besides providing ATP, NADH and FADH2, Acetyl-CoA is the main products of FAO process [27]. Acetyl-CoA serves as the intersection to many metabolic pathways and contributes to the fatty acid synthesis and TCA cycle [28]. Moreover, the acetyl-CoA is the required acetyl donor for lysine acetylation and thereby links metabolism and epigenetics [29]. Some reports have shown that Nanog can be epigenetically modified. Nanog promoter can be modified by methylation [30] and acetylation [31] while Nanog mRNA can be modified by m6A-demethylation under certain conditions [32]. In embryonic stem cells, acetyltransferase P300 could acetylate histone H3K27 and H3K56, and promoted the expression of NANOG, OCT4 and SOX2 to maintain pluripotency [31]. We identified that P300 and H3K27 acetylation was enhanced in serum-deprived CRC cells, data further showed that FAO induced P300 expression to accelerate H3K27 acetylation of Nanog promoter in serum-deprived CRC cells.

Our data showed that ACLY expression was induced by FAO under serum deprivation, which contributed to the synthesize of acetyl-CoA in the cytoplasm and nucleus. However, the exact mechanism needs to be further studied. In fact, abnormal expression or activity of ACLY has been found in hepatic steatosis, dyslipidemia, diabetes and types of cancers, including colon cancer [33]. Its overexpression correlates with poor prognosis in cancer [34] as it could facilitate proliferation, metastasis and stemness of cancer cells. In addition, considerable interest has been addressed in ACLY as a target for anticancer drugs [35, 36]. Our study broadened the function of ACLY by demonstrating that ACLY could induce dormancy of CRC cells via regulating Nanog.

However, some issues in our study were remained to be solved. Firstly, the mechanism of FAO regulating ACLY expression needs further exploration. Then, more kinds of cellular dormancy models are needed to verify our findings.

In conclusion, our study put forward a novel mechanism of cellular dormancy in CRC cells. Data clearly showed that dormant CRC cells upregulated the expression of Nanog through a FAO-ACLY-dependent pathway. FAO increased ACLY expression to promote the synthesis of acetyl-CoA, which was transferred by P300 to accelerate H3K27 acetylation of Nanog promoter. Then, Nanog upregulation promoted the transcription of P21 and P27 to mediate the dormancy of CRC cells. This novel axis represents a connection between metabolism, epigenetic reprogramming and cellular dormancy in CRC.

## Supplementary information

Supplementary information

Figure S1

Figure S2

Figure S3

Figure S4

Figure S5

Figure S6

Figure S7

Figure S8

Figure S9

Figure S10

Figure S11

ACTIN

Table S1

Table S2

Table S3

aj-checklist

cddis-author-contribution-form

## Data Availability

All data generated or analyzed during this study are included in this published article [and its supplementary information files].
