# Supplementary material for: Nanog mediated by FAO/ACLY signaling induces cellular dormancy in colorectal cancer cells
Source: Cell Death Dis. 2022 Feb 17;13(2):159. doi: 10.1038/s41419-022-04606-1 (PMC8854412; doi:10.1038/s41419-022-04606-1)
Supplement: Supplementary file 1 — Supplementary information [file 41419_2022_4606_MOESM1_ESM.docx]

**Nanog mediated by FAO/ACLY signaling** **induces cellular dormancy in colorectal cancer cells**

Meng Zhang^1, 2^*, Ruyi Peng^1, 2^*, Haizhou Wang^1, 2^*, Zhenwei Yang^1, 2^, Hailin Zhang^1, 2^, Yangyang Zhang^1, 2^, Meng Wang^1, 2^, Hongling Wang^1, 2^, Jun Lin^1, 2^, Qiu Zhao^1, 2#^, Jing Liu^1, 2#^

**Author Affiliations**

^1^Department of Gastroenterology, Zhongnan Hospital of Wuhan University, Wuhan 430071, China;

^2^Hubei Clinical Center & Key Lab of Intestinal & Colorectal Diseases, Wuhan 430071, China;

*These authors contributed equally to this work.

^#^**Corresponding authors: Jing Liu (**[liujing_GI@whu.edu.cn](mailto:liujing_GI@whu.edu.cn)**)** or **Qiu Zhao (**[qiuzhao@whu.edu.cn](mailto:qiuzhao@whu.edu.cn)**)**, Department of Gastroenterology, Zhongnan Hospital of Wuhan University, No. 169, Donghu Road, Wuchang District, Wuhan 430071, Hubei Province, China. Phone: +86 13971605755 or +86 18971622466. Fax: +86 27 67812892.

**Conflict of interests**

The authors declare that they have no known competing financial interests or personal relationships that could have appeared to influence the work reported in this paper.

**Supplementary Information**

**Summary**

Figure S1-11 and the corresponding figure legends.

Actin: RT-PCR using β-actin as internal reference in experiments with glycolysis inhibitor 2-DG.

Table S1: Sequences of primers for PCR.

Table S2: Sequences of primers for CHIP-PCR.

Table S3: Sequences of siRNA.

**Supplementary Figures and Figure legends**

**
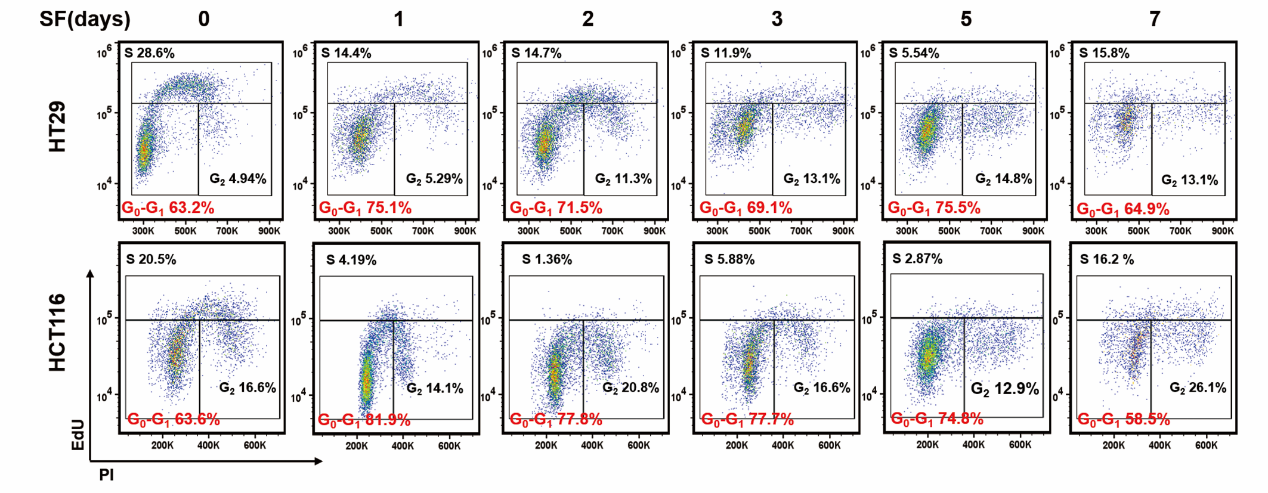
**

**Figure S1.** Cell cycle analysis of CRC cells by serum deprivation for different time. CRC cells were cultured in serum withdrawal for 0, 1, 2, 3, 5, 7days, cell cycle was analyzed by FCM. SF: Serum free; FCM: Flow cytometry. Data represented at least 3 independent experiments.


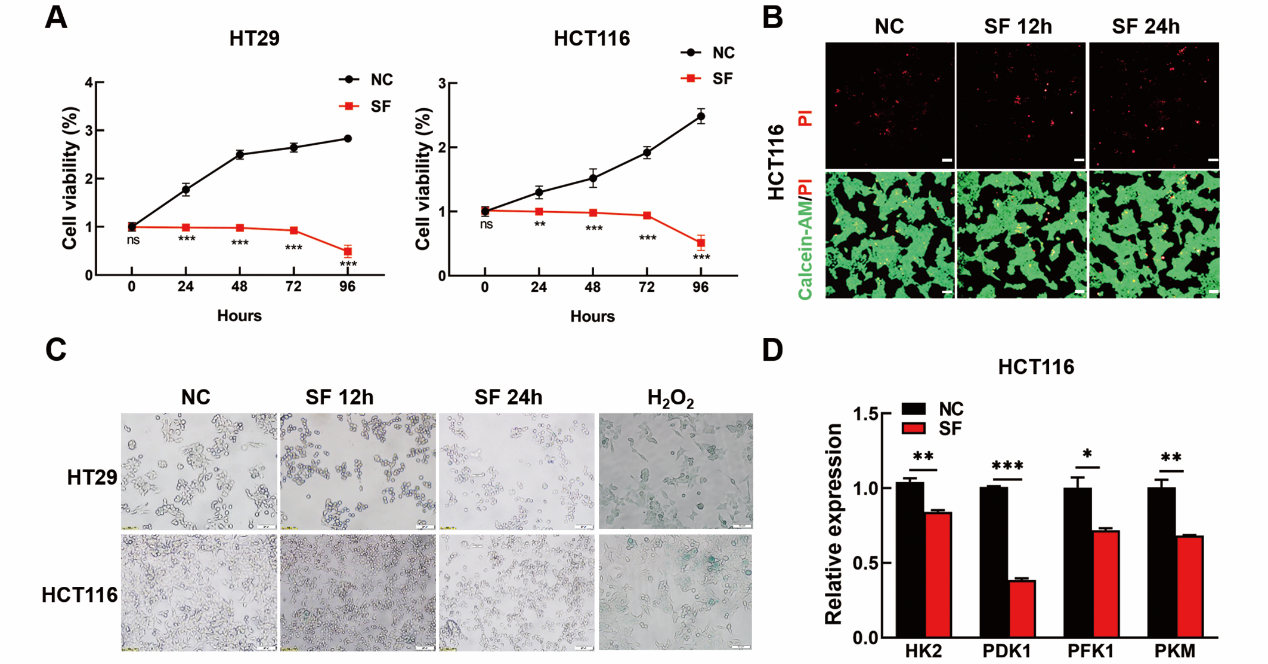


**Figure S2.** **(A**) HT29 and HCT116 were cultured in serum free conditions (SF) or normal conditions (NC). Cell viability was analyzed with CCK8 from 0 to 96h. **(B)** Calcein-AM/PI staining of serum deprived CRC cells. HCT116 cells were cultured in serum free conditions for 12 hours or 24 hours. Cells in normal conditions were used as controls. Living cells were stained by Calcein-AM (green) while dead cells were visualized by PI (red). The stained cells were observed under a fluorescence microscope and photographed. Scale bar = 50μm. **(C)** CRC cells were normal cultured or serum deprived for 12 hours or 24 hours. SA-β gal staining was conducted. CRC cells treated with 200μM H2O2 for 2.5h were used as positive control. Scale bar = 50μm. **(D)** The mRNA expression of genes (*HK2*, *PDK1*, *PFK1* and *PKM*) involved in glucose catabolism in NC and SF HCT116 cells was analyzed by PCR. Data are shown as the means ± s.e.m., n = 3; ns: no statistical significance, ***P* < 0.01, *** *P* < 0.001. Data shown represented three independent experiments.

**
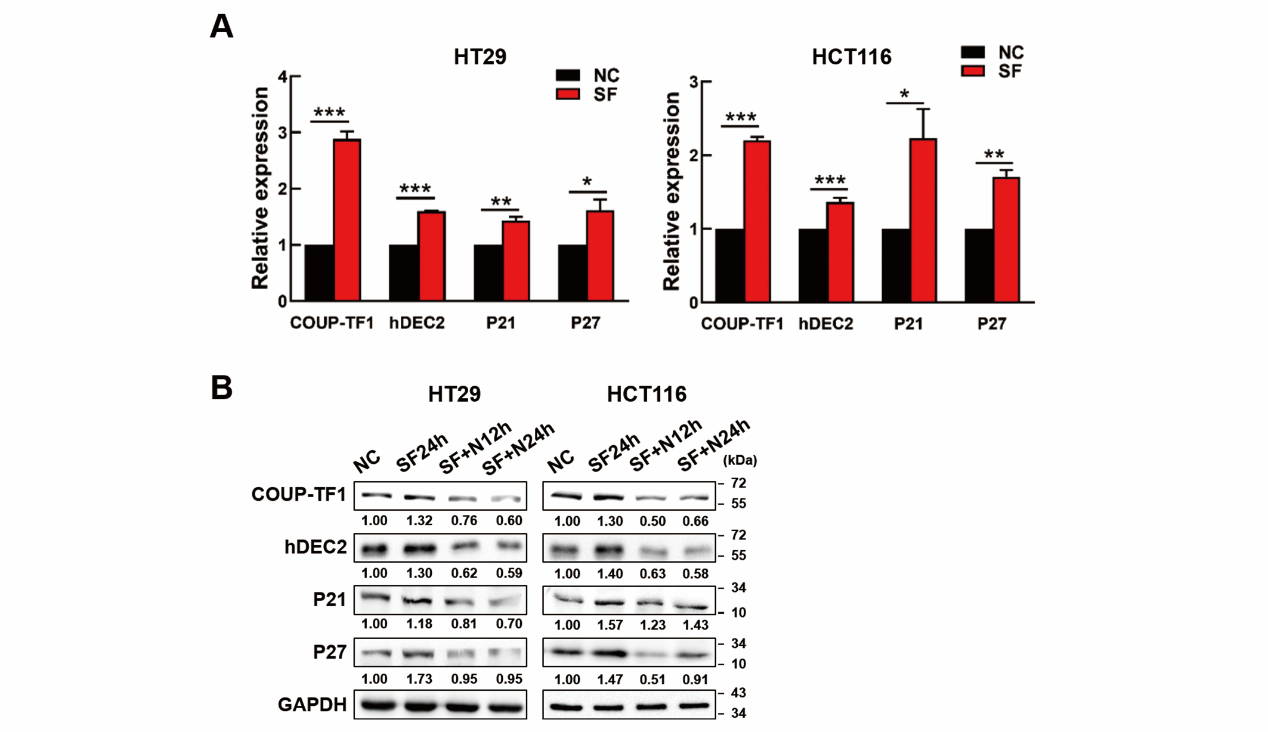
**

**Figure S3. (A)** The mRNA expression of *COUP-TF1*, *hDEC2*, *P21* and *P27* in NC and SF CRC cells was analyzed by PCR. **(B)** COUP-TF1, hDEC2, P21 and P27 protein levels of CRC cells in serum deprived or serum replenished condition. Data are shown as the means ± s.e.m., n = 3; ns: no statistical significance, ***P* < 0.01, *** *P* < 0.001. Data shown represented three independent experiments.


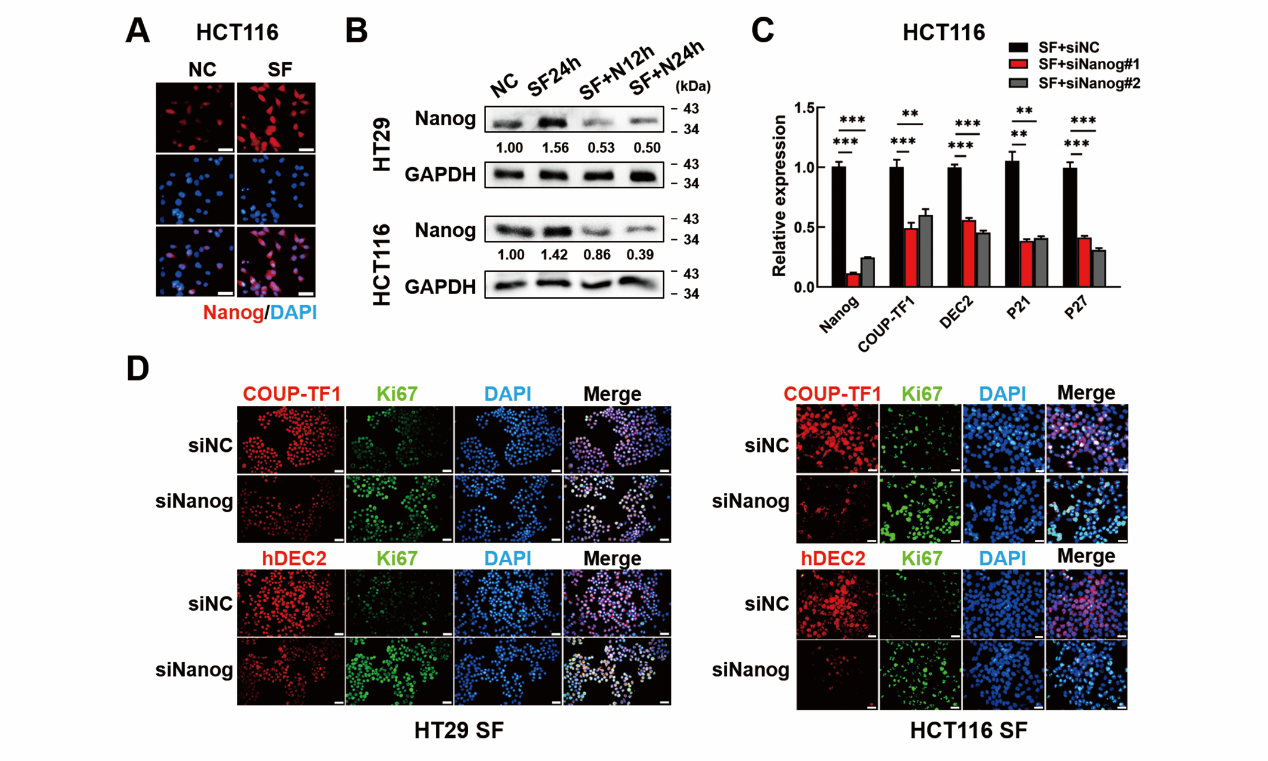


**Figure S4. (A)** Immunofluorescence staining of Nanog from normal cultured or serum deprived HCT116 cells. **(B)** Nanog protein level of CRC cells in serum deprived or serum replenished condition. **(C)** Nanog knockdown decreased the mRNA expression of *COUP-TF1*, *hDEC2*, *P21* and *P27* in SF HCT116 cells. Data are shown as the means ± s.e.m., ***P* < 0.01, *** *P* < 0.001. **(D)** Immunofluorescence staining of COUP-TF1 or hDEC2 and Ki67 by Nanog inhibition in SF CRC cells. The stained cells were visualized by a fluorescent microscope. Scale bar = 20μm. Data shown represented three independent experiments.


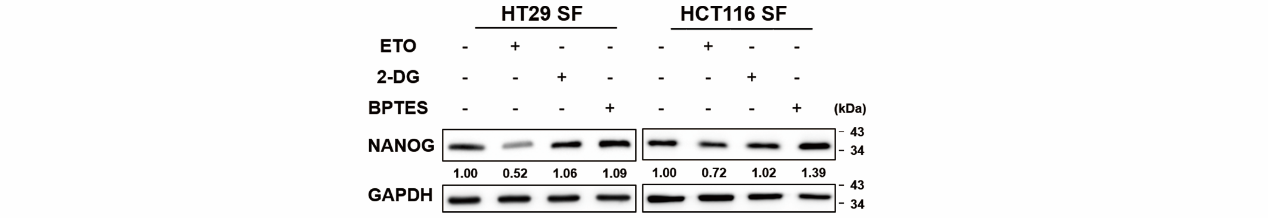


**Figure S5.** ETO could block Nanog expression in serum-deprived CRC cells while 2-DG and BPTES could not. The CRC cells were serum deprived and treated with 100μM ETO, 10μM 2-DG or 10μM BPTES for 24 hours. Then, the cells were tested for Nanog expression by Western blotting.


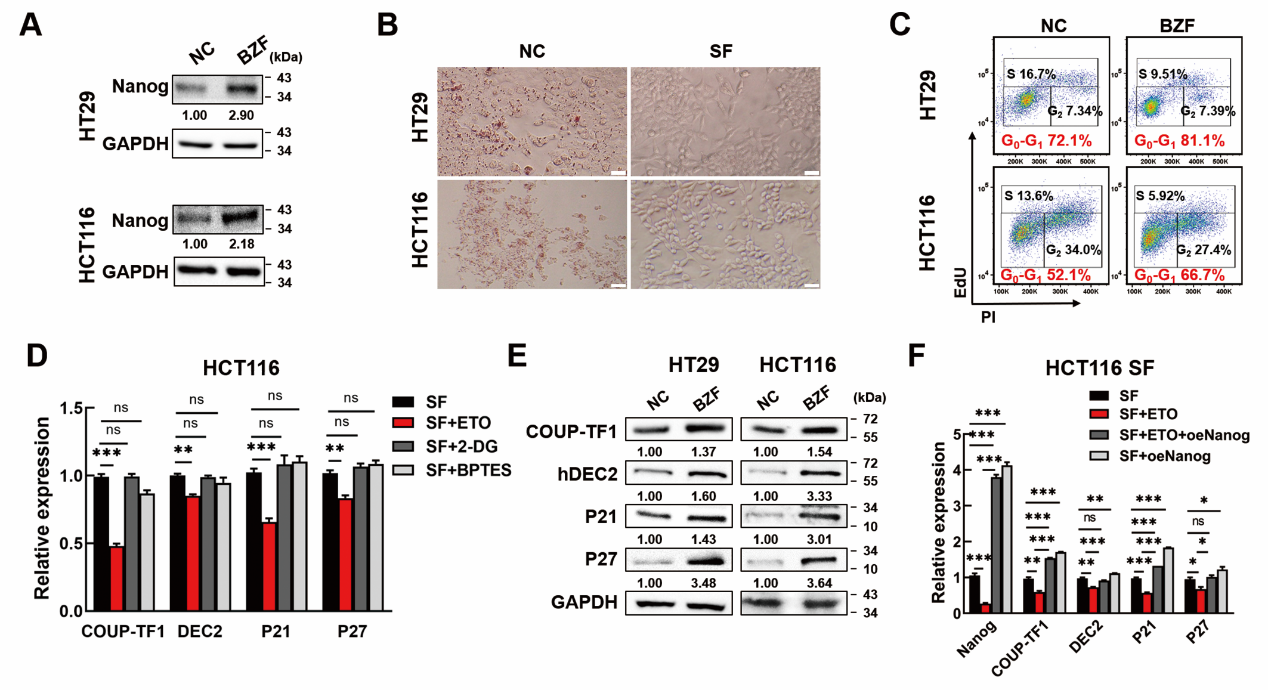


**Figure S6. (A)** CRC cells were treated with 10μM BZF or solvent DMSO for 24h, Nanog protein level was examined by Western blotting. **(B)** Oil red O staining showed lipid droplets in normal culture and SF CRC cells. Scale bar = 20μm. **(C)** Cell cycle analyze of CRC cells treated with 10μM BZF or solvent DMSO. **(D)** ETO could block the expression of *COUP-TF1*, *hDEC2*, *P21* and *P27* in SF HCT116 cells while 2-DG and BPTES could not. **(E)** BZF treatment improved the protein levels of COUP-TF1, hDEC2, P21 and P27 in SF CRC cells. **(F)** The blockage of dormancy markers by ETO could be reversed by Nanog overexpression in SF HCT116 cells. DMSO: dimethyl sulfoxide, ETO: etomoxir, FAO inhibitor; 2-DG: 2-Deoxy-D-glucose, glycolysis inhibitor; BPTES: glutaminase inhibitor; BZF: bezafibrate, FAO agonist. Data are shown as the means ± s.e.m., n = 3; ns: no statistical significance, **P* < 0.05, ***P* < 0.01, *** *P* < 0.001. Data shown represented three independent experiments.

**
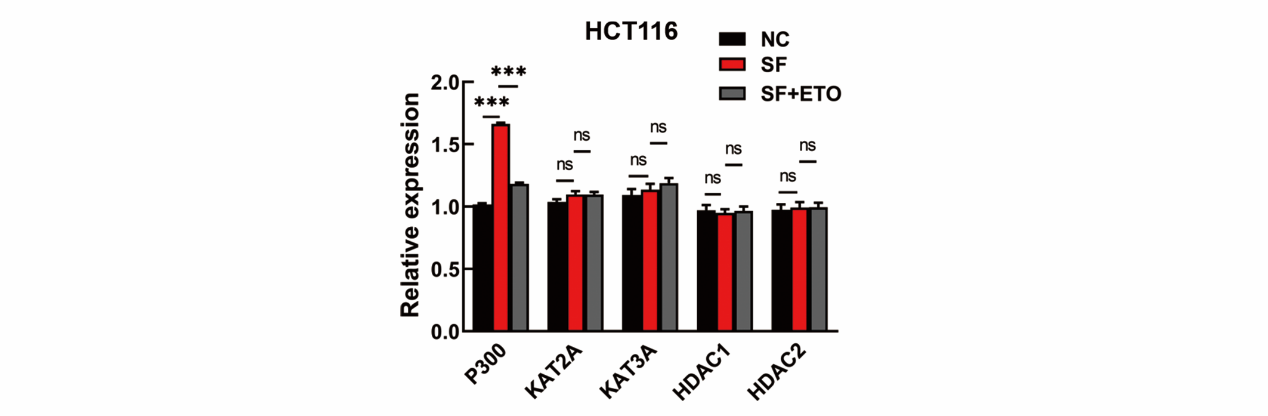
**

**F****igure S7.** The expression levels of histone acetyltransferases (*P300, KAT2A, KAT3A*) and histone deacetylases (*HDAC1, HDAC2*) were examined by PCR in normal HCT116 cells, in SF HCT116 cells and in SF HCT116 cells treated with ETO. Data are shown as the means ± s.e.m., n = 3; ns: no statistical significance, *** *P* < 0.001. Data shown represented three independent experiments.

**
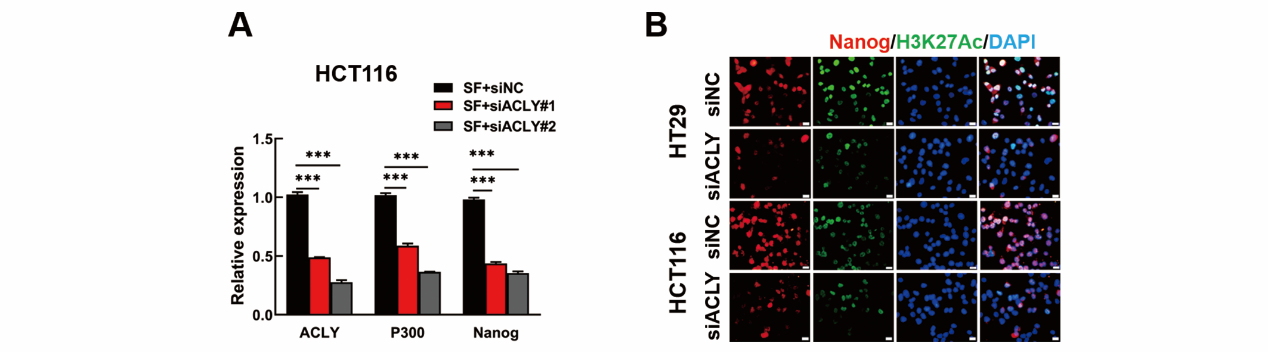
**

**Figure S8. (A)** ACLY knockdown decreased the expression of *P300* and *Nanog*. Serum deprived HCT116 cells were transfected with ACLY siRNAs or negative control siRNAs. The expression of ACLY, P300 and Nanog was analyzed by PCR. **(B)** Immunofluorescence staining of Nanog and H3K27Ac by ACLY inhibition in serum deprived CRC cells. The cells were examined under the fluorescent microscope. Scale bar = 10μm. Data are shown as the means ± s.e.m., n = 3; *** *P* < 0.001. Data shown represented three independent experiments.

**
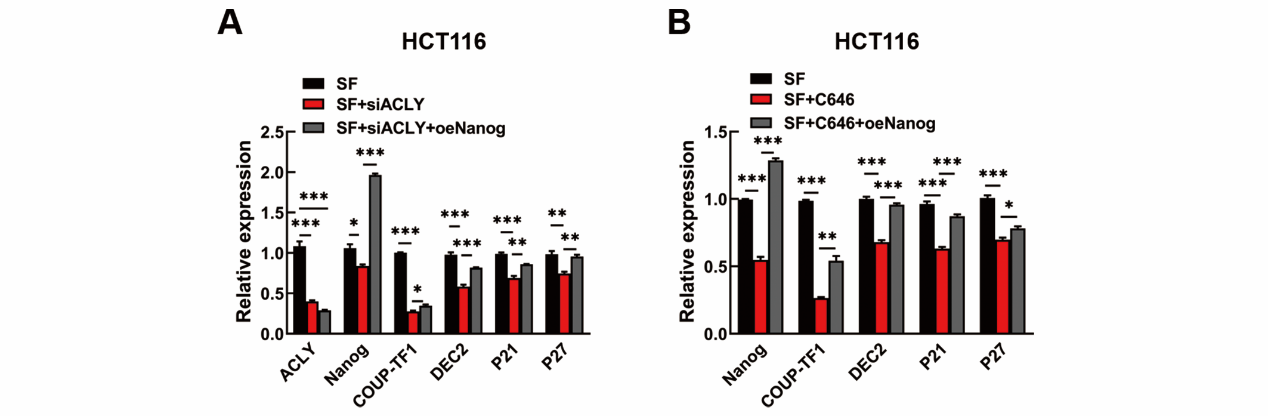
**

**Figure S9.** **(A)** Dormancy markers could be inhibited by ACLY inhibition and reversed by Nanog overexpression in SF HCT116 cells. **(B)** Dormancy markers could be inhibited by P300 inhibition and reversed by Nanog overexpression in SF HCT116 cells. Data are shown as the means ± s.e.m., n = 3; **P* < 0.05, ***P* < 0.01, *** *P* < 0.001. Data shown represented three independent experiments.


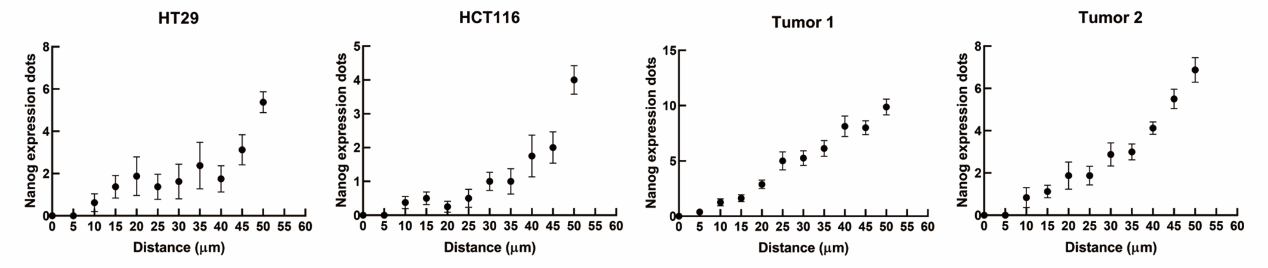
 **Figure S10.** Nanog expression dots were counted according to the distance to blood vessels (n=8) in HT29, HCT116, Tumor 1, Tumor 2.


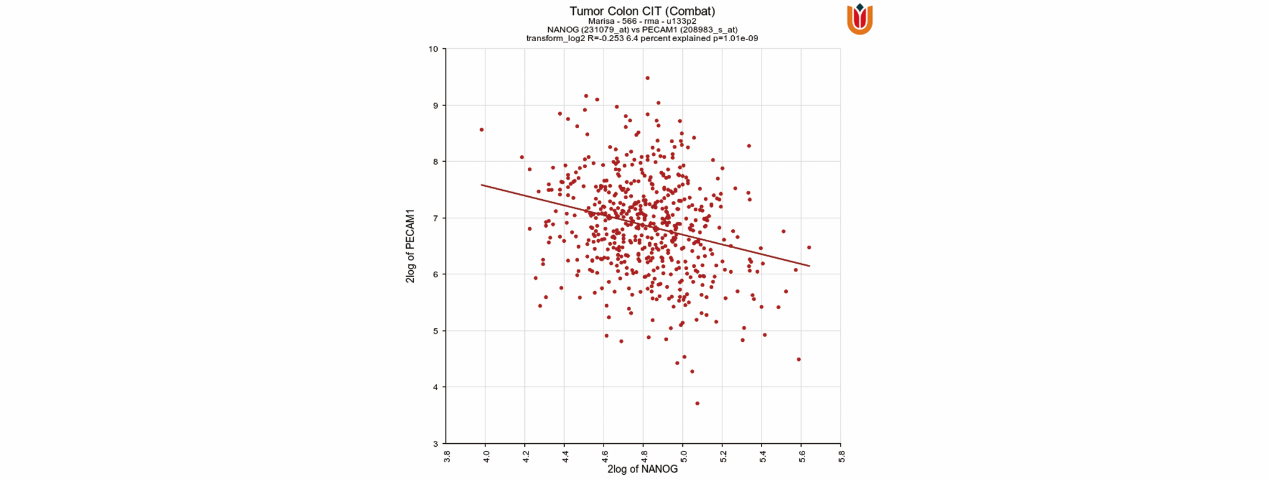


**Figure S11.** Pearson correlation analysis between CD31 and Nanog in colon cancer tissues (R = -0.253; *P* = 1.01e-09). The colon cancer dataset GSE39582 (n=566) was chosen to analyze the correlation between CD31 and Nanog in R2 website (<https://hgserver1.amc.nl/cgi-bin/r2/main.cgi>).


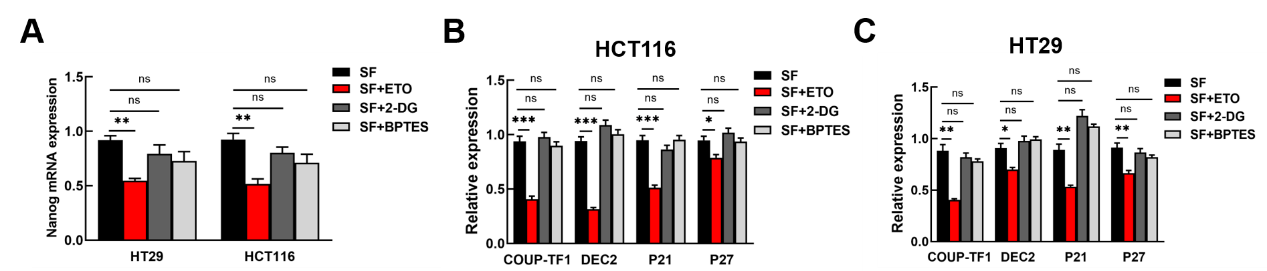


**Actin.** RT-PCR using β-actin as internal reference in experiments with glycolysis inhibitor 2-DG.
