# Supplementary figures and images for: Nanog mediated by FAO/ACLY signaling induces cellular dormancy in colorectal cancer cells
Source: Cell Death Dis. 2022 Feb 17;13(2):159. doi: 10.1038/s41419-022-04606-1 (PMC8854412; doi:10.1038/s41419-022-04606-1)

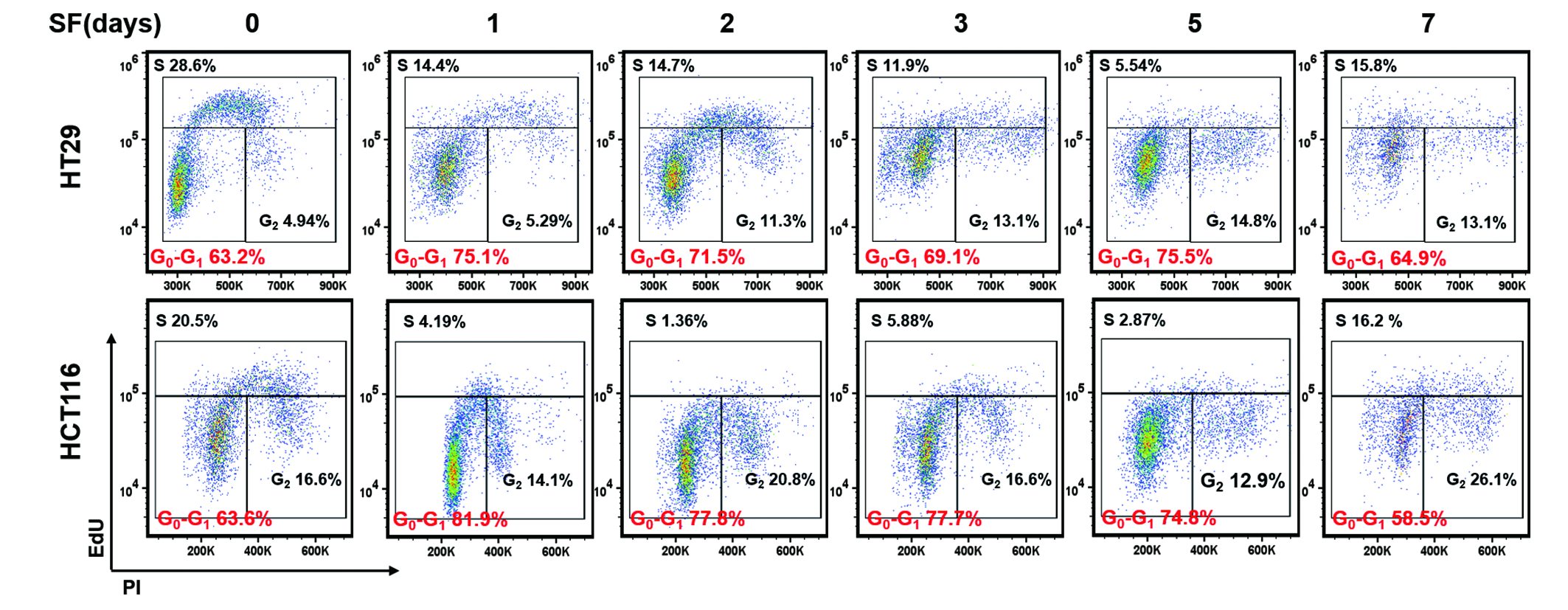

Supplement: Supplementary file 2 — Figure S1 [file 41419_2022_4606_MOESM2_ESM.tif]

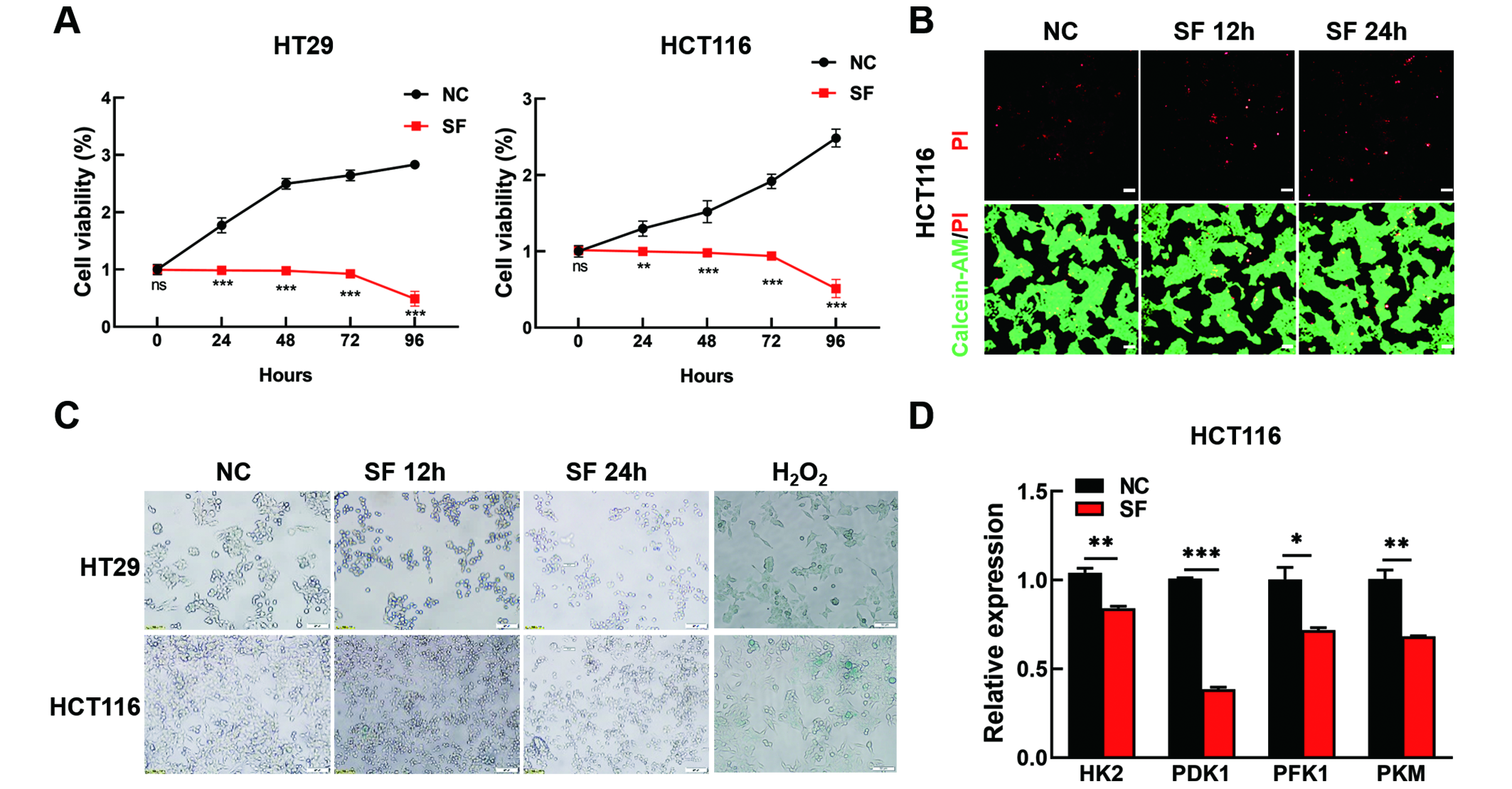

Supplement: Supplementary file 3 — Figure S2 [file 41419_2022_4606_MOESM3_ESM.tif]

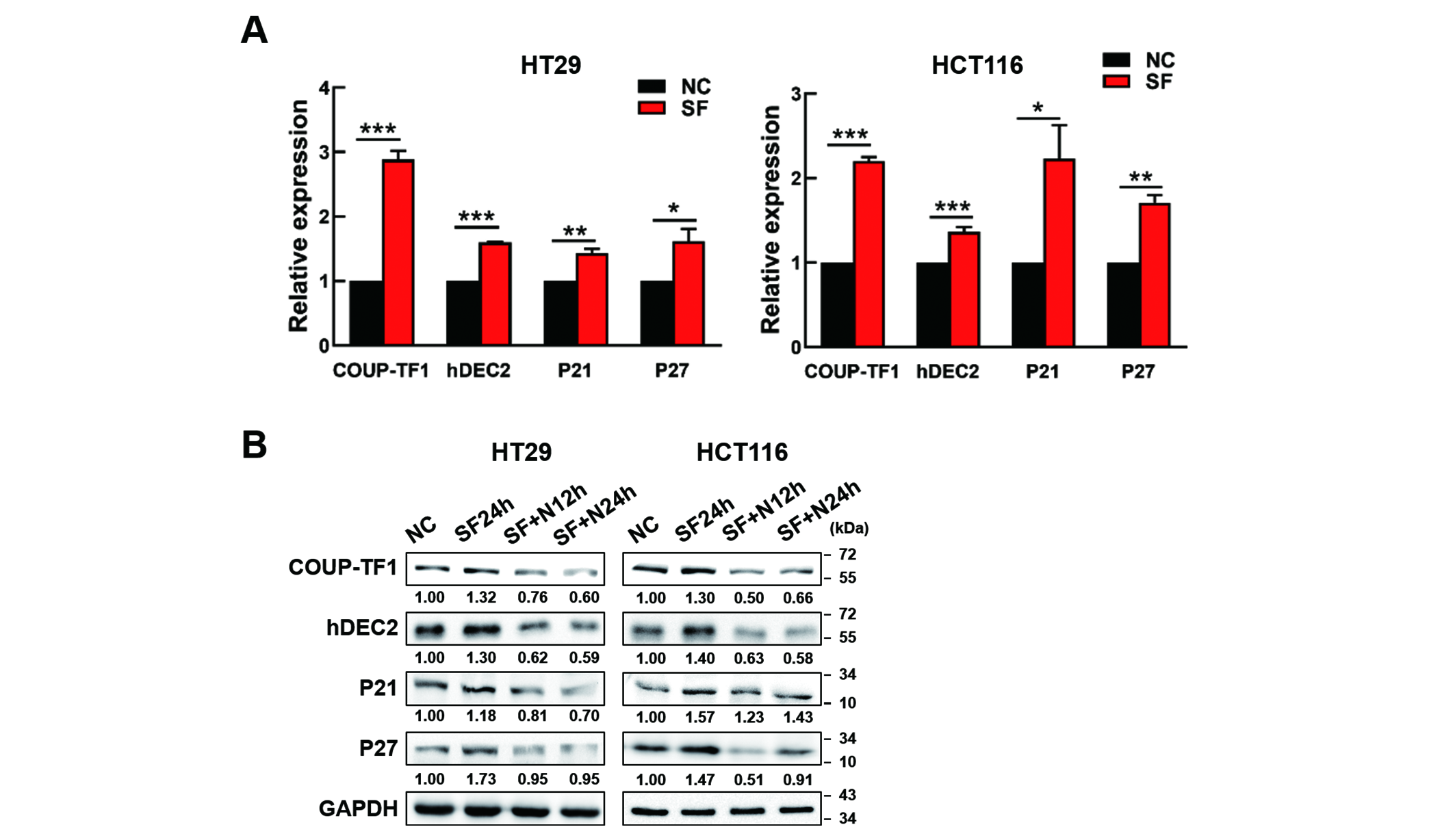

Supplement: Supplementary file 4 — Figure S3 [file 41419_2022_4606_MOESM4_ESM.tif]

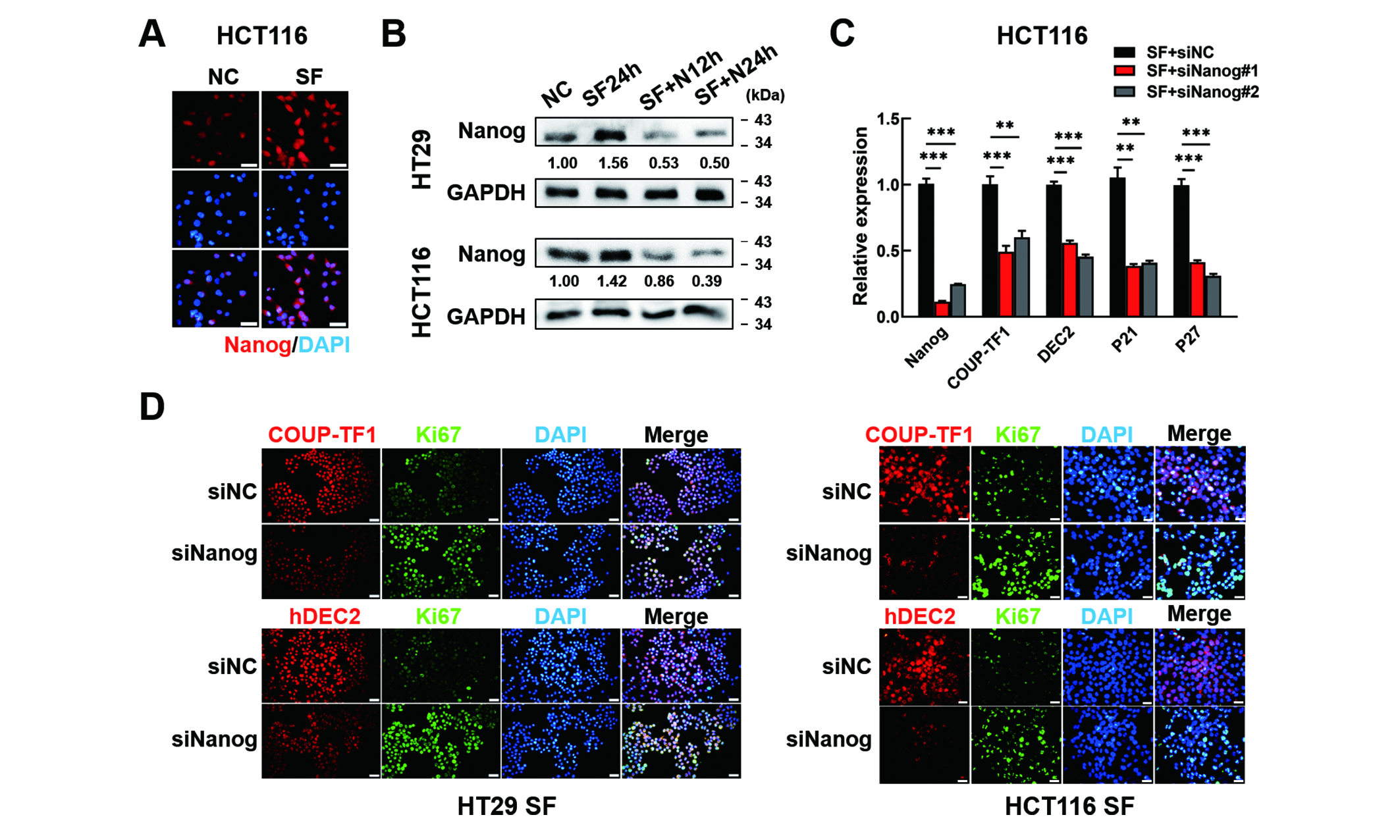

Supplement: Supplementary file 5 — Figure S4 [file 41419_2022_4606_MOESM5_ESM.tif]

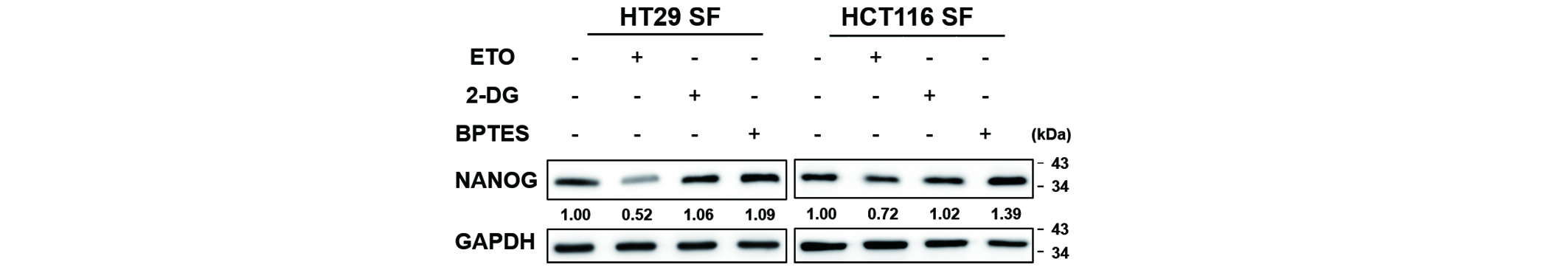

Supplement: Supplementary file 6 — Figure S5 [file 41419_2022_4606_MOESM6_ESM.tif]

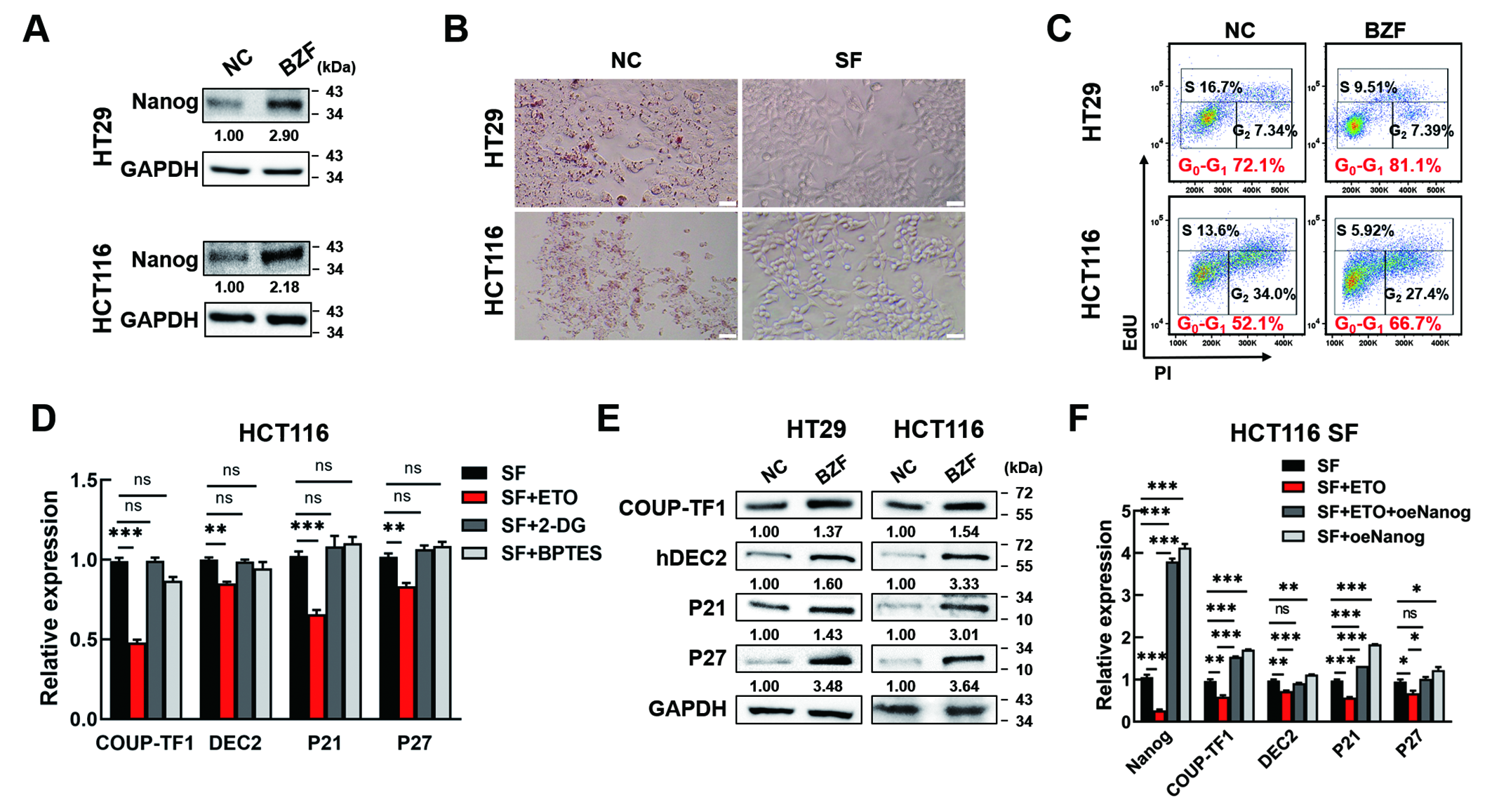

Supplement: Supplementary file 7 — Figure S6 [file 41419_2022_4606_MOESM7_ESM.tif]

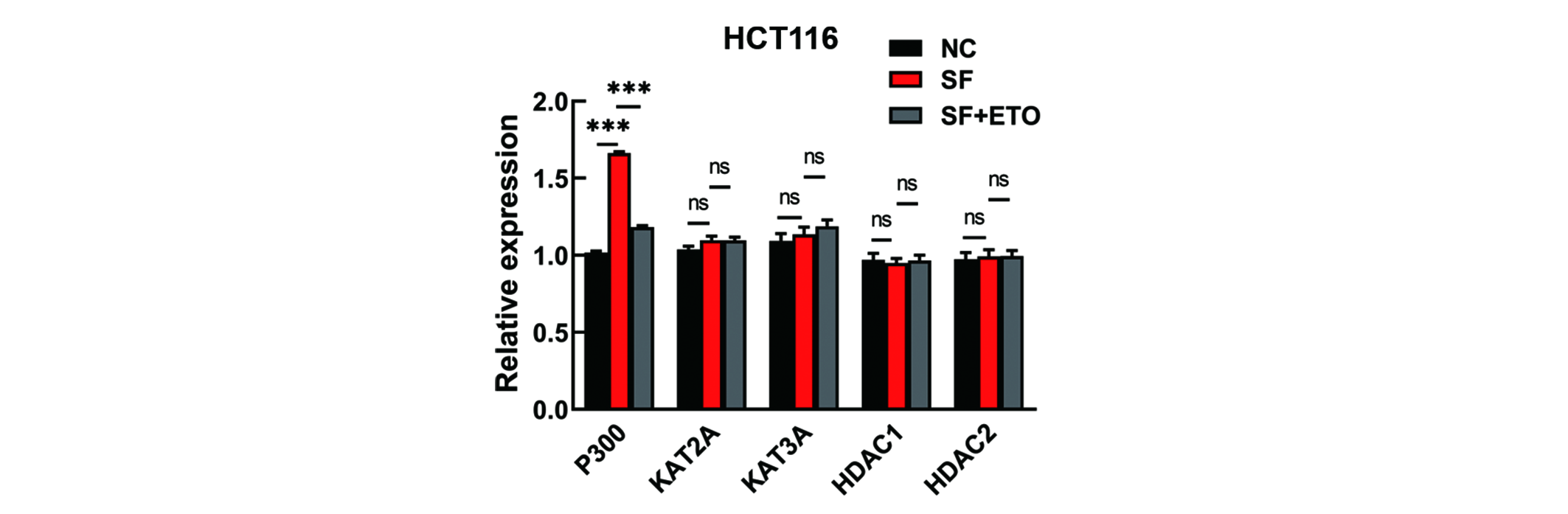

Supplement: Supplementary file 8 — Figure S7 [file 41419_2022_4606_MOESM8_ESM.tif]

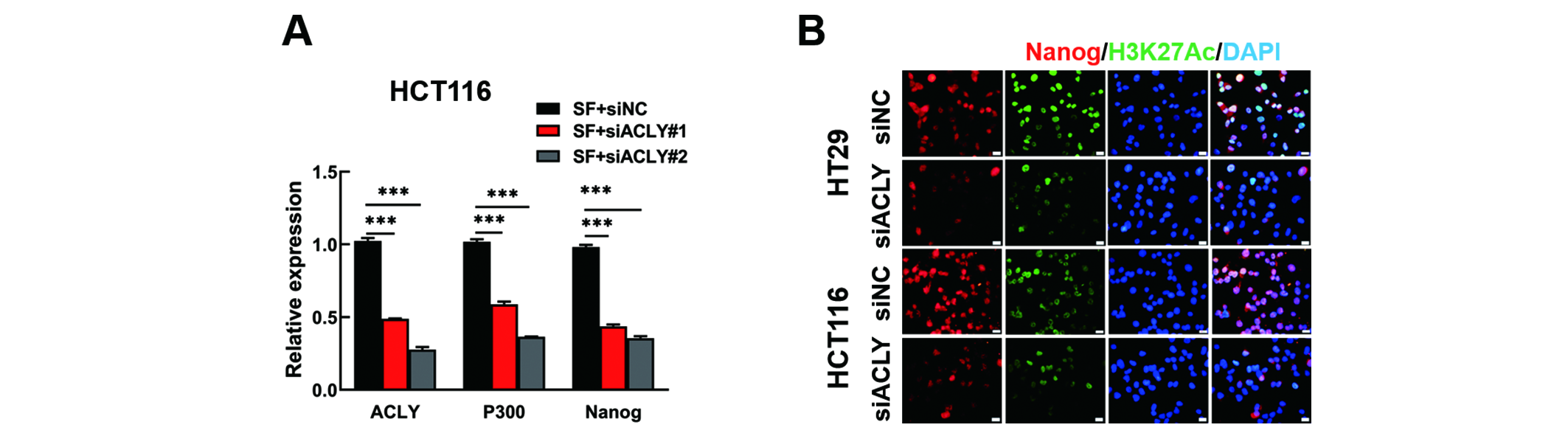

Supplement: Supplementary file 9 — Figure S8 [file 41419_2022_4606_MOESM9_ESM.tif]

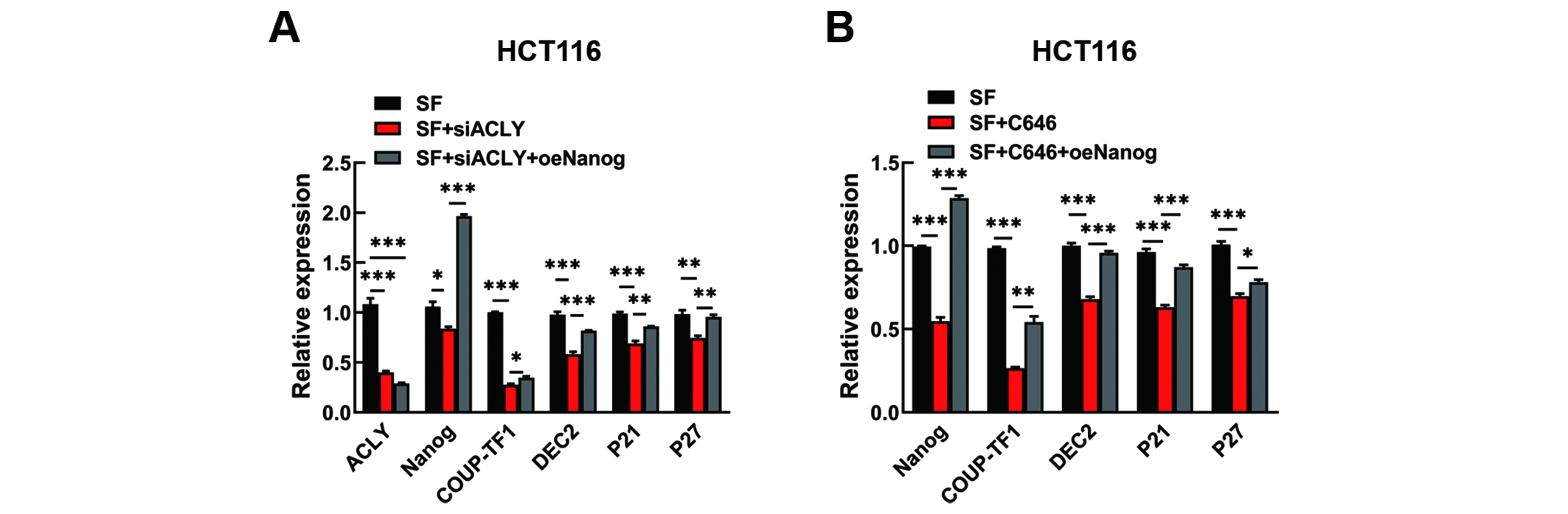

Supplement: Supplementary file 10 — Figure S9 [file 41419_2022_4606_MOESM10_ESM.tif]

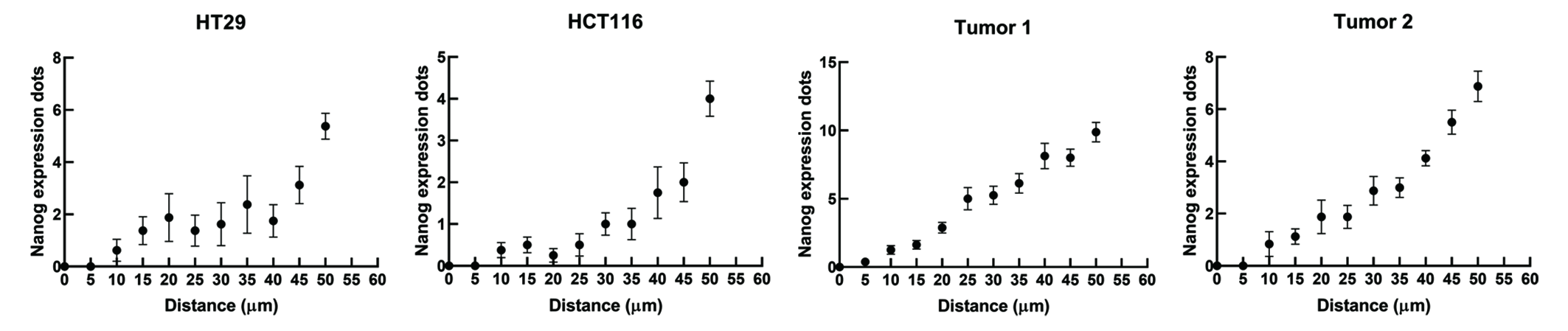

Supplement: Supplementary file 11 — Figure S10 [file 41419_2022_4606_MOESM11_ESM.tif]

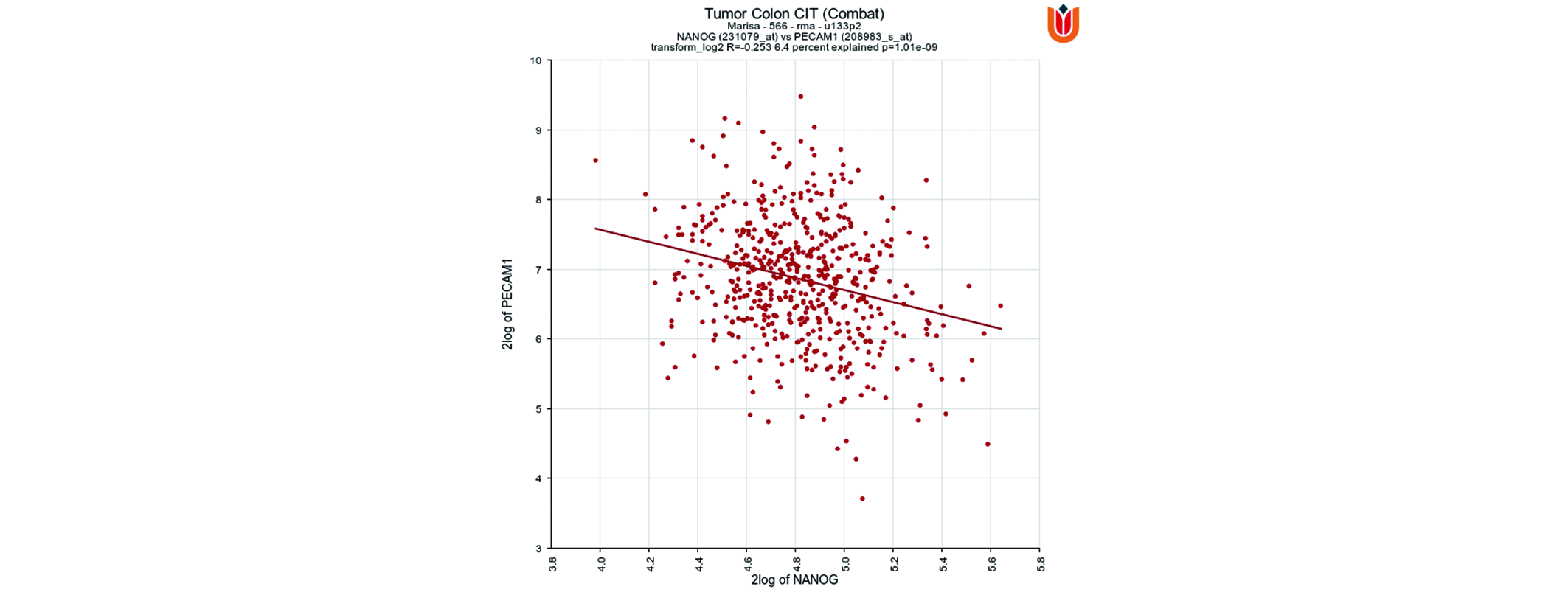

Supplement: Supplementary file 12 — Figure S11 [file 41419_2022_4606_MOESM12_ESM.tif]

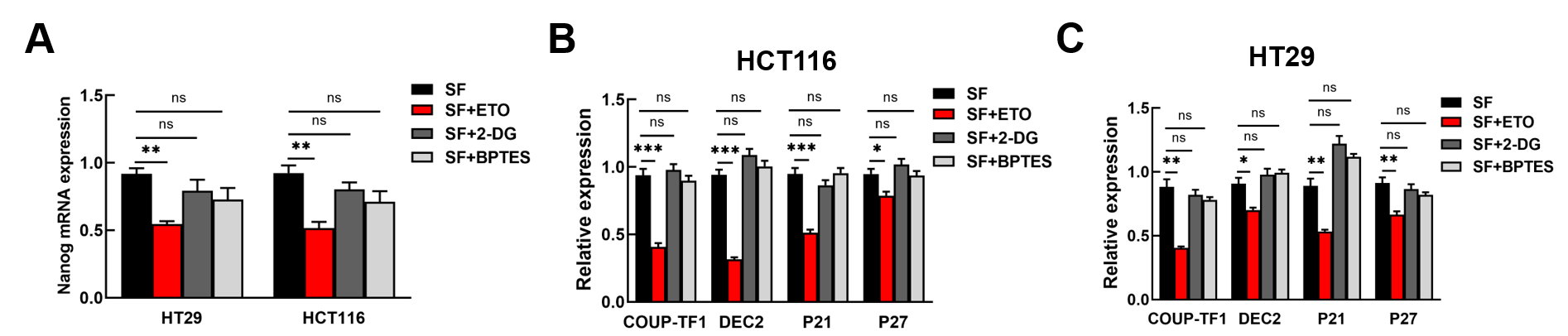

Supplement: Supplementary file 13 — ACTIN [file 41419_2022_4606_MOESM13_ESM.tif]
