# Supplementary material for: Nanog mediated by FAO/ACLY signaling induces cellular dormancy in colorectal cancer cells
Source: Cell Death Dis. 2022 Feb 17;13(2):159. doi: 10.1038/s41419-022-04606-1 (PMC8854412; doi:10.1038/s41419-022-04606-1)
Supplement: Supplementary file 18 — cddis-author-contribution-form [file 41419_2022_4606_MOESM18_ESM.pdf]

DECLARATION OF CONTRIBUTIONS TO ARTICLE

ADMC

Manuscript Number: CDDIS-21-2316R

Journal Name: *Cell Death & Disease* (the 'Journal')

Proposed Title of the Contribution: Author contribution statement (the 'Contribution)

Author(s): Meng Zhang, Ruyi Peng, Haizhou Wang, Zhenwei Yang, Hailin Zhang, Yangyang Zhang, Meng Wang, Hongling Wang, Jun Lin, Qiu Zhao and Jing Liu. (the 'Authors')

For all *CDDis* articles, each person named as an author in the published version must be able to show he or she has contributed substantially to the article.

Authorship credit should be based on 1) substantial contributions to conception and design, acquisition of data, or analysis and interpretation of data; 2) drafting the article or revising it critically for important intellectual content; and 3) final approval of the version to be published. Authors should meet conditions 1, 2 and 3.

Any person who cannot be shown to have made a substantial contribution to the article cannot be listed as an author in the final version. The name of any person who is deemed to have made a minor contribution can, however, appear in the Acknowledgments section of the article.

Please complete the table below to indicate the contributions of all named authors to the manuscript.

Author Full Name:	Specification of Contribution to the Manuscript:
Meng Zhang	Designed the experiments, performed the experiments and wrote the manuscript.
Ruyi Peng	Performed the experiments and wrote the manuscript.
Haizhou Wang	Analyzed the results and revised the manuscript.
Zhenwei Yang	Assembled figures.
Hailing Zhang	Assembled figures.
Yangyang Zhang	Assembled figures.
Meng Wang	Collected human specimens and performed IHC experiments.
Hongling Wang	Collected human specimens and performed IHC experiments.
Jun Lin	Collected human specimens and performed IHC experiments.
Qiu Zhao	Supervised experiments and checked the manuscript.
Jing Liu	Designed the experiments, supervised experiments and checked the manuscript.

Please complete the table below to indicate the contributions of all named authors to the figures.

Figure 1:

Zhang M and Peng RY generated the data, Wang HZ analyzed the results and Yang ZW assembled the figure.

Figure 2:

Zhang M and Peng RY generated the data, Wang HZ analyzed the results and Yang ZW assembled the figure.

Figure 3:

Zhang M and Peng RY generated the data, Wang HZ analyzed the results and Zhang HL assembled the figure.

Figure 4:

Zhang M and Peng RY generated the data, Wang HZ analyzed the results and Zhang HL assembled the figure.

Figure 5:

Zhang M and Peng RY generated the data, Wang HZ analyzed the results and Zhang YY assembled the figure.

Figure 6:

Zhang M and Peng RY generated the data, Wang HZ analyzed the results and Zhang YY assembled the figure.
For Figure 7, Peng RY, Wang M, Wang HL and Lin J generated the data, Wang HZ analyzed the results and Zhang HL assembled the figure.

Signed for and on behalf of the Author(s):	Print Name:	Date:
刘静 Jing Liu	Jing Liu	December 25, 2021
